# Inferring Connectivity Range in Submerged Aquatic Populations (*Ruppia* L.) Along European Coastal Lagoons From Genetic Imprint and Simulated Dispersal Trajectories

**DOI:** 10.3389/fpls.2018.00806

**Published:** 2018-06-13

**Authors:** Ludwig Triest, Tim Sierens, Dimitris Menemenlis, Tom Van der Stocken

**Affiliations:** ^1^Ecology and Biodiversity Research Group, Plant Biology and Nature Management, Vrije Universiteit Brussel, Brussels, Belgium; ^2^Jet Propulsion Laboratory, California Institute of Technology, Pasadena, CA, United States

**Keywords:** isolation-by-distance (IBD), genetic divergence, dispersal modeling, seagrass, microsatellite

## Abstract

Coastal salt- and brackish water lagoons are unique shallow habitats characterized by beds of submerged seagrasses and salt-tolerant *Ruppia* species. Established long-term and large-scale patterns of connectivity in lagoon systems can be strongly determined by patterns of nearshore and coastal currents next to local bird-mediated seed dispersal. Despite the importance of dispersal in landscape ecology, characterizing patterns of connectivity remains challenging in aquatic systems. Here, we aimed at inferring connectivity distances of *Ruppia cirrhosa* along European coastal lagoons using a population genetic imprint and modeled dispersal trajectories using an eddy-resolving numerical ocean model that includes tidal forcing. We investigated 1,303 individuals of 46 populations alongside subbasins of the Mediterranean (Balearic, Tyrrhenian, Ionian) and the Atlantic to Baltic Sea coastline over maximum distances of 563–2,684 km. Ten microsatellite loci under an autotetraploid condition revealed a mixed sexual and vegetative reproduction mode. A pairwise *F*_ST_ permutation test of populations revealed high levels of historical connectivity only for distance classes up to 104–280 km. Since full range analysis was not fully explanatory, we assessed connectivity in more detail at coastline and subbasin level using four approaches. Firstly, a regression over restricted geographical distances (300 km) was done though remained comparable to full range analysis. Secondly, piecewise linear regression analyses yielded much better explained variance but the obtained breakpoints were shifted toward greater geographical distances due to a flat slope of regression lines that most likely reflect genetic drift. Thirdly, classification and regression tree analyses revealed threshold values of 47–179 km. Finally, simulated ocean surface dispersal trajectories for propagules with floating periods of 1–4 weeks, were congruent with inferred distances, a spatial Bayesian admixed gene pool clustering and a barrier detection method. A kinship based spatial autocorrelation showed a contemporary within-lagoon connectivity up to 20 km. Our findings indicate that strong differentiation or admixtures shaped historical connectivity and that a pre- and post LGM genetic imprint of *R. cirrhosa* along the European coasts was maintained from their occurrence in primary habitats. Additionally, this study demonstrates the importance of unraveling thresholds of genetic breaks in combination with ocean dispersal modeling to infer patterns of connectivity.

## Introduction

The ability and limitation of dispersal influence colonization success and hence patterns of landscape connectivity (Cain et al., 2000), and present an important component of a species' ecological resilience (Les et al., 2003). The interaction of dispersal with phenology, the spatial distribution of dispersal barriers and suitable habitat, shape the distribution of a species. Wide distributions such as known in many aquatic plants (Cook, 1996) are attributed to efficient dispersal mechanisms and dispersal vectors that allow for transport over vast spatial scales. As a consequence, direct observations and accurate measurements of dispersal distances in riverine, coastal and lacustrine landscapes are difficult. Genetic assessments present the most straightforward way to assess realized connectivity or gene flow, which is the result of dispersal, recruitment success and selection pressures (Sanford and Kelly, 2011). Modeling approaches in contrast allow to simulate likely dispersal trajectories and provide information on the nature and scale of potential connectivity. Comparing genetic and physical connectivity estimates can be useful especially for discerning the main modes of dispersal as well as relevant time scales (White et al., 2010). While most ocean current assessments are in the range of days to months, genetic structure reflects realized gene flow over multiple generations (Benzie, 1999). Genetic structure of the allelic diversity therefore can provide information on long-lasting dispersal barriers. These discontinuities could correspond to relevant contemporary barriers, e.g., opposing currents at large scale but also human constructions such as marinas and dikes, impacting the connectivity of coastal lagoons and preventing propagule inflow or outflow. Empirical data generally reflects a complex and multifaceted contemporary genetic structure that results from various demographic, environmental, and historical processes that species experienced (Meirmans, 2015). Both contemporary ocean currents and historical isolation due to Pleistocene sea-level fluctuations have been shown to influence the genetic structure in marine and coastal populations (Hernawan et al., 2017). Throughout the Pleistocene (about 2.6 Ma-12 ka) sea level fluctuated following repeated glacial-interglacial cycles. Since the Last Glacial Maximum (LGM, ca. 23–19 ka BP), sea level rose *ca*. 115–130 m up to the present level (Lambeck et al., 2014), strongly changing the configuration of coastlines and water bodies, the availability of habitats, and affecting ocean circulation.

*Ruppia* L. is a cosmopolitan genus of submerged macrophytes, far related to seagrass families such as *Cymodoceceaea* and *Posidoniaceae*. It grows in shallow lagoons and coastal wetlands where they can tolerate high fluctuations in salinity and water level (Mannino et al., 2015). Coastal lagoons are ecotones between terrestrial, freshwater and marine ecosystems (Basset and Abbiati, 2004), usually separated from the ocean by a temporary sand barrier with only one or a few inlets, and with varying tidal mixing and salinities. They support a rich biodiversity, and shallow habitats of submerged vegetation act as spawning grounds for marine fish, invertebrates and present resting areas for many species of migratory birds (Aliaume et al., 2007). In lagoons with severe salinity and water level fluctuations, *Ruppia* species are almost the only submerged macrophytes withstanding these dynamic conditions. In these unique habitats, *Ruppia* forms large monospecific beds of great importance for harboring invertebrates and algae (Verhoeven, 1980).

Since the brackish water lagoons are often temporary open to or closed off from the sea, dispersal certainly plays a significant role in structuring the distribution of *Ruppia* species. *Ruppia cirrhosa* (Petagna) Grande (also named Ruppia spiralis L. ex Dumortier) can occur in saltmarshes, salinas, estuaries, and coastal lakes. *Ruppia cirrhosa* populations can co-occur with other seagrasses such as *Zostera* and with marine macroalgae (Triest and Sierens, 2013). Their establishment might have originated from drifting propagules transported by sea currents. Seed production on flowering and drifting shoots might increase the dispersal distance. The seeds of several seagrasses generally are too heavy to travel long distances by water currents alone (Orth et al., 1994) but floating reproductive shoots were frequently observed at the sea surface. Orth et al. (1994) suggested that long-distance dispersal (LDD) and colonization of distant habitats may be achieved via these floating plants and fragments. Fully ripened *Ruppia* fruits may detach and sink readily to the bottom. However, larger shoot fragments with rhizomes and flowering/fruiting parts regularly detach and survive when floating (Triest and Sierens, 2013). Detached vegetative parts of the plants remain free-floating with immediate root initiation upon reaching the bottom (Verhoeven, 1979). The most probable means of a seagrass colonizing nearby shores is through the transportation of seeds on floating and fruiting shoots. Shoots may have become detached through stochastic disturbances such as stormy weather or the activities of waterfowl feeding on sediment-burrowing invertebrates. Though the dispersal of seagrass propagules occurs mainly through abiotic transport by wave action, tidal flow and ocean currents, migrating and local free-moving water birds may disperse propagules over great distances, as known for many non-marine aquatic plants (Les et al., 2003). Long-distance dispersal (LDD), however, is supposed to be rare and gene flow caused by present day dispersal would play a minor role as compared to past events (Clausen et al., 2002). Nevertheless, while the majority of seeds may have been discarded by birds when they arrive, the few that are still trapped in the intestinal system of the birds may represent rare but important dispersal events (Figuerola and Green, 2002).

In the European part of the Mediterranean at least 15 cpDNA haplotypes of *Ruppia* can be distinguished and characterized, representing a diversity hotspot (Triest and Sierens, 2014). *Ruppia cirrhosa* shows a West–East differentiation for maternal cpDNA. It was hypothesized that birds as vectors of maternal cpDNA markers did not homogenize the genetic structure, but the presence of few scattered isolated and rare haplotypes reflected a thin tail of occasional LDD events (Triest and Sierens, 2013). Sea currents were discussed as a potential dispersal vector at broad geographic scales for maternal haplotypes of *R. cirrhosa*, the most marine *Ruppia* representative, thereby resembling other seagrasses in open lagoons (Triest and Sierens, 2013). Throughout Europe, *R. cirrhosa* is far more widespread and locally abundant in a much wider variety of coastal habitats than *Ruppia maritima* L. (Triest and Sierens, 2014). The number of *Ruppia* species and their distribution is largely unknown (Short et al., 2007) thereby leading to misunderstandings of species identity (den Hartog and Kuo, 2006; Ito et al., 2010; Triest and Sierens, 2014). Likewise, identification of its evolutionary units at a continental scale is complicated by morphologically variable (Triest and Symoens, 1991) and hybridized populations, especially in lagoons of the Mediterranean (Ito et al., 2013; Triest and Sierens, 2014; Martinez-Garrido et al., 2016). A higher level of haplotypic diversity in *Ruppia* populations was reported for the Iberian Peninsula (Triest and Sierens, 2010) and 12 microsatellite markers developed for *R. maritima* aided in identifying ancient introgressed and recent hybrids with *R. cirrhosa* (Triest and Sierens, 2015), whereas *R. cirrhosa* could be clearly distinguished from other *Ruppia* species using a molecular toolkit of 24 microsatellites (Triest et al., 2018). Pollination takes place at the water surface (epihydrophilous) over restricted distances. This lack of pollen flow over large distances is in contrast with abiotic pollination in many terrestrial species (Ellstrand, 2014).

European (from Baltic Sea to Mediterranean) *R. cirrhosa* showed a differential colonization pattern since the LGM on basis of chloroplast sequences. Only one haplotype group (C) occurs along the Atlantic, North Sea and Baltic Sea, whereas a diverse haplotype group (B) occurs as either pure populations or mixed with haplogroup C in the Mediterranean (Triest and Sierens, 2014). LDD or repeated multiple short distance dispersal (SDD) happened during the northward expansion along the Atlantic, North Sea, and Baltic coastlines from a narrow maternally related source. On the contrary, the Mediterranean Sea and its different basins still might be representative of long-lasting dispersal and re-colonization patterns (except the more recently flooded Northern Adriatic). CpDNA haplotypes of *R. cirrhosa* were informative of the shallow phylogenies (Triest et al., 2018) and maternal seed dispersal (Triest and Sierens, 2014). However, nuclear DNA (microsatellites) will be more performant for determining the genetic connectivity over various distance classes within each basin (or coastline) under recent dynamics. It can be expected that strongly changed coastlines (i.e., recently colonized) contain higher levels of relatedness (kinship) than the more or less “stable” coastlines that remained largely confined within Mediterranean subbasins during and after the LGM. Physically connected populations along the coastlines are considered to reveal low or no genetic structure. However, present-day genetic structure could still reflect ancient genetic structure at a time when sea level was far below the actual level, and the shape of the coastline and geographical barriers were different.

In this study, we aim to (1) analyse the global imprint of *R. cirrhosa* population genetic information across Europe and (2) estimate the connectivity of *R. cirrhosa* populations at the level of coastlines and subbasins, using genetic, statistical and dispersal modeling approaches. The null hypothesis refers to a random process without spatial structure. Therefore, we assessed populations of *R. cirrhosa* in Europe, considering various geographic areas (Baltic—North Sea—Atlantic; Balearic Sea; Tyrrhenian Sea; and the East Mediterranean basin with Adriatic Sea, Ionian Sea and Aegean Sea), at distances ranging from a few to hundreds of kilometers within subbasins. We combined nuclear microsatellite based genetic structure and connectivity analysis with physical connectivity assessments to discern whether local dispersal or LDD could explain observed population structures. More specifically, we aim to test for potential genetic breaks at different spatial scales, namely for (1) local contemporary dispersal and inferred connectivity range within hydrologically connected sites, and (2) historical connectivity within subbasins at a regional scale. Simulated ocean dispersal trajectories and patterns of regional connectivity inferred from genetic data are compared with other species and discussed for potential interference with bird-mediated processes. In particular, we propose to conduct alternative approaches: breakpoint analysis, classification and regression trees (CRT) for threshold determination, and a combination of Bayesian population structure and barrier detection with dispersal trajectory modeling of a common submerged aquatic plant in coastal lagoons.

## Materials and methods

### Materials

#### Study sites and plant materials

*Ruppia cirrhosa* plants were collected during the period 2006–2012 in 46 coastal water bodies from Europe (Table 1). The considered biogeographic regions are the coastlines of the Baltic Sea, North Sea, Atlantic, Balearic, Tyrrhenian, Adriatic, Ionian, and Aegean subbasins. In each site, up to 30 individual shoots were collected along a 30 m transect, except for few smaller stands. A total of 1,303 individual shoots were dried on silica gel and a reference herbarium for each population was deposited at BRVU (Herbarium of Vrije Universiteit Brussel, transferred in 2018 to BR, Botanic Garden, Meise, Belgium). Chloroplast haplotype diversity and nuclear ITS characterization of these same *R. cirrhosa* populations were described in Triest and Sierens (2009, 2010, 2013, 2014) and ensured a correct species identity for samples of the current study. A nomenclatural taxon name shift from *R. cirrhosa* to *R. spiralis* could be applicable (Ito et al., 2017) for all populations considered in this study.

**Table 1:** Summary statistics of population genetic variables for *Ruppia cirrhosa* populations along coastlines of Europe.

**Region/Population code**	**Locality**	**Latitude**	**Longitude**	***N***	**G**	**R**	***A***	***A*_e_**	***H*_O_**	***H*_E_**	***F*_i_**	***p***
1 Europe grand mean				1,303	955	0.80	18.1	5.9	0.533	0.744	0.570	< 0.001
Baltic-Atlantic				330	205	0.64	9.1	3.4	0.435	0.624	0.610	< 0.001
2_GER	Hiddensee, Kloster, Enddorn	54.596	13.138	24	18	0.74	3.9	2.4	0.435	0.455	0.315	< 0.001
3_GER	Hiddensee, Kloster, Schwedenhagen	54.584	13.125	28	25	0.89	4.9	2.8	0.562	0.568	0.298	< 0.001
4_GER	Hiddensee, Vitte, Furt	54.545	13.114	30	23	0.76	4.7	2.7	0.452	0.549	0.511	< 0.001
5_GER	Wustrow, Rerik	54.103	11.611	30	6	0.17	3.5	2.5	0.450	0.483	0.375	< 0.001
6_NL	Zeeland	51.68	4.017	26	4	0.12	1.6	1.5	0.400	0.335	−0.324	0.185
7_FR	Nord PDC, Platier d'Oye	51.007	2.082	30	29	0.97	2.0	1.7	0.341	0.305	0.284	< 0.001
8_FR	Nord PDC, Le Fort Vert 1	50.986	1.942	38	33	0.86	2.2	1.6	0.270	0.281	0.425	< 0.001
9_FR	Aquitaine, Audange, Graveyron 2	44.689	−1.042	56	14	0.24	2.6	2.1	0.593	0.435	−0.238	0.003
10_FR	Aquitaine, Audenge, Certes 1	44.677	−1.017	53	38	0.71	4.8	2.6	0.488	0.547	0.468	< 0.001
11_FR	Aquitaine, Le Verdon-sur-Mer, Marais du Conseiller 1	45.541	−1.072	15	15	1.00	3.2	2.5	0.451	0.471	0.41	< 0.001
Balearic Sea				417	349	0.83	13.4	4.7	0.568	0.718	0.526	< 0.001
12_FR	Camargue, Trou de l'Oie	43.363	4.814	21	21	1.00	5.4	3.2	0.570	0.644	0.437	< 0.001
13_FR	Camargue, Le Capouillet	43.362	4.810	29	27	0.93	6.5	3.8	0.728	0.671	0.175	< 0.001
14_FR	Carnon	43.552	3.995	27	24	0.92	6.4	3.8	0.624	0.664	0.381	< 0.001
15_SP	Aiguemolls NP	42.233	3.115	31	24	0.80	3.9	2.1	0.412	0.478	0.512	< 0.001
16_SP	Estartit Old River	42.029	3.190	29	29	1.00	4.1	2.7	0.562	0.608	0.429	< 0.001
17_SP	Estartit New pond	42.031	3.193	28	27	0.96	3.7	2.5	0.587	0.567	0.322	< 0.001
18_SP	Delta de l'Ebre	40.686	0.854	25	17	0.67	4.6	2.8	0.665	0.589	0.155	< 0.001
19_SP	Albufera NP Valencia, Marina	39.346	−0.315	26	5	0.16	2.1	1.9	0.380	0.388	0.156	0.233
20_SP	St Pola Canal	38.184	−0.613	38	36	0.95	6.5	3.6	0.618	0.644	0.306	< 0.001
21_SP	Roquetas de Mar (Alboran Subbasin)	36.716	−2.644	24	12	0.48	2.6	2.1	0.409	0.406	0.31	< 0.001
22_SP	Mallorca, Sa Coma	39.567	3.372	28	23	0.81	2.8	1.7	0.310	0.324	0.439	< 0.001
23_SP	Es Grau, saltmarsh transect	39.946	4.265	22	21	0.95	5.5	3.2	0.584	0.627	0.405	< 0.001
24_SP	Es Grau, Albufera	39.947	4.263	29	26	0.89	6.0	3.5	0.609	0.655	0.392	< 0.001
25_IT	Oristano, Stagno Istai	39.970	8.461	30	29	0.97	6.1	3.5	0.615	0.661	0.38	< 0.001
26_IT	Oristano, Santa Giusta lagoon	39.872	8.609	30	28	0.93	4.9	3.6	0.527	0.581	0.401	< 0.001
Tyrrhenian Sea				188	114	0.60	10.4	5.4	0.573	0.743	0.480	< 0.001
27_IT	Porto Corallo lagoon	39.435	9.618	29	19	0.64	3.6	2.3	0.432	0.409	0.272	< 0.001
28_IT	Cagliari (W-lagoon) salinas	39.181	9.024	26	16	0.56	5.2	2.8	0.697	0.615	0.085	0.025
29_IT	Chia, Su Giudeu	38.889	8.868	27	27	1.00	6.5	3.7	0.546	0.676	0.484	< 0.001
30_IT	Castiglione della Pescaia, Badiola	42.782	10.940	30	15	0.56	2.6	2.0	0.321	0.366	0.379	< 0.001
31_IT	Borgo Grappa, Circeo NP	41.388	12.920	46	23	0.49	4.7	3.5	0.751	0.652	−0.03	0.271
32_IT	Trapani, Location1	37.860	12.485	10	8	0.78	2.8	2.2	0.721	0.510	−0.261	0.005
33_IT	Trapani, Location2	37.869	12.486	20	6	0.26	2.9	2.7	0.558	0.491	0.051	0.590
Adriatic–Ionian				368	287	0.78	11.1	5.0	0.556	0.704	0.485	< 0.001
34_IT	Grado, NR Valle Cavanata, Italy	45.715	13.476	29	13	0.43	3.9	2.6	0.662	0.548	−0.037	0.487
35_SLO	Secovlje salina NP	45.492	13.608	30	30	1.00	5.0	2.6	0.443	0.489	0.429	< 0.001
36_SLO	Secovlje salina NP, small salina	45.528	13.609	28	27	0.96	4.2	2.7	0.466	0.494	0.379	< 0.001
37_GR	Arta, Logarou	39.013	20.924	22	20	0.90	3.9	2.6	0.530	0.541	0.328	< 0.001
38_GR	Arta, Logarou, inner side of dike	39.017	20.929	30	21	0.69	4.4	3.4	0.614	0.596	0.243	< 0.001
39_GR	Arta, Logarou, Koronissiu	39.033	20.849	21	21	1.00	4.7	2.8	0.625	0.535	−0.054	0.205
40_GR	Arta, Logarou, Lake Tsoukalio	39.061	20.874	22	6	0.24	2.3	2.2	0.683	0.437	−0.605	< 0.001
41_GR	Messolonghi, third lagoon	38.333	21.429	29	22	0.75	5.4	3.5	0.817	0.667	−0.012	0.776
42_GR	Messolonghi, sixth lagoon	38.332	21.432	15	7	0.43	3.3	3.0	0.913	0.629	−0.381	< 0.001
43_GR	Achaia, Lake Prokopos, northern transect	38.005	21.288	25	22	0.88	4.5	2.6	0.420	0.508	0.467	< 0.001
44_GR	Achaia, Lake Prokopos, southern transect	37.996	21.282	29	29	1.00	4.1	2.9	0.637	0.533	−0.027	0.381
45_GR	Ilia, Lake Kotychi, northern transect	38.160	21.386	24	23	0.96	5.8	3.3	0.530	0.620	0.446	< 0.001
46_GR	Ilia, Lake Kotychi, southern transect	38.151	21.387	29	26	0.89	6.0	3.5	0.477	0.609	0.519	< 0.001
47_GR	Monolimni	40.807	26.021	35	20	0.56	3.7	2.5	0.425	0.461	0.366	< 0.001

*Population codes (GER, Germany; NL, The Netherlands; FR, France; SP, Spain; IT, Italy; SLO, Slovenia; GR, Greece), locality, decimal coordinates, sample size (N), genet size (G), clonal richness (R), mean number of alleles (A), effective number of alleles (A_e_), observed heterozygosity (H_O_), expected heterozygosity (H_E_), individual inbreeding coefficient (F_i_) with p-value*.

### Methods

#### DNA extraction and microsatellite amplification

Genomic DNA was extracted from leaf tissue using the E.Z.N.A. SP plant DNA Mini Kit (Omega bio-tek, Norcross, GA, USA) as in Triest and Sierens (2010). Microsatellite loci for *Ruppia* were isolated from a microsatellite-enriched genomic library following an enrichment procedure with Dynabeads (Glenn and Schable, 2005). We used RsaI and SspI as restriction enzymes and repeat motifs AG, TG, GATA, and GACA. Out of 96 sequences for each species, 18 from *R. cirrhosa* (haplotype C, ITS-B, Estartit, Spain) and 27 from a hybrid *Ruppia* (haplotype B, ITS-B, Valencia, Spain) were identified as potential for designing primers with Primer3 (http://primer3.wi.mit.edu/). From these tested primer pairs, we selected polymorphic loci presenting at least 10 di-, tri- and tetranucleotide repeats flanked by more than 10 nucleotides between primers region and motifs (Table S1). Additional microsatellite loci for *R. cirrhosa* (Estartit, Spain) were obtained by 100 bp paired-end sequencing on an Illumina Hiseq by Macrogen (Seoul, republic of Korea). Generated reads were first converted from FASTQ format to FASTA using.NET Bio 1.01 (https://bio.codeplex.com). Subsequently, QDD2.1 (Meglécz et al., 2010) was used to identify microsatellites and design primers. In *R. cirrhosa*, a total of 21.01 million reads yielded 127,903 reads containing a microsatellite insert of at least five repeats of which 11,407 were suitable for primer design. Discarding loci with compound SSR's and high penalty values, and selection based on fragment length and number of repeats, 36 primers for *R. cirrhosa* were selected for synthesis. PCR products were tested for amplification and polymorphism by electrophoresis on a Qiaxcel (Qiagen). Eight polymorphic loci designed from *R. cirrhosa* were retained for this study: *RC3, RCS5, RCS8, RCS9, RCS27, RM3, RM12, RM26*. One microsatellite (*RMB15*) of European *R. maritima* was cross-amplified from Triest and Sierens (2015). All primers developed for *R. sinensis* were tested but only *RUMR4* (Yu et al., 2009) was retained as a diagnostic marker. Five additional microsatellite primers recently developed from S. Iberian samples (Martínez-Garrido et al., 2014) and used in a regional case-study by Martinez-Garrido et al. (2016) were tested *a posteriori* and gave good amplification for *R. cirrhosa*. On basis of allele lengths, the primers for each locus were labeled and amplified (Ta = 57°C) in a multiplexed reaction (Table S1) using the QIAGEN Multiplex PCR Plus kit. We developed a multiplexed set of 10 loci for *R. cirrhosa* out of 24 microsatellites (Triest et al., 2018). PCR products were run on ABI3730XL sequencer (Macrogen, Seoul, Korea) and fragments were analyzed with GeneMarker V2.20 (SoftGenetics LLC®). Experimental genotyping avoided bias in interpretation (allele sizes, stutter peaks, artifact ghost amplicons) by testing each primer separately prior to screening in a multiplex, including repeats, randomization of populations on each plate and an independent scoring.

#### Population genetic variables

Similar multilocus genotypes (MLGs) were obtained from GenAlex v6.5 (Peakall and Smouse, 2012) to calculate the number of genets (G) per population. Because of the tetraploid nature of the species, most conventional methods and software packages (designed for diploids) could not be used. Therefore, we focused on suitable methods for autotetraploids in SPAGeDi v1.5 (Hardy and Vekemans, 2002; Vekemans and Hardy, 2004) and further statistical testing. All alleles belonging to the same locus were considered within an autotetraploid setting, though unbalanced heterozygotes could not be considered. This methodological approach underestimates these extra heterozygosity combinations over four homologous chromosomes and could overestimate inbreeding levels. At population level we estimated the clonal diversity (*R* = 1–G/1–N), mean number of alleles (*A*), effective number of alleles (*A*_e_), allelic richness (*A*_r_ at *k* = 20), mean allele sizes, gene diversities (observed and expected heterozygosities *H*_O_ and *H*_E_), individual inbreeding coefficient (*F*_i_ with *p*-value). The mean of the abovementioned variables and the global ANOVA-based *F*-statistics and *R*-statistics (1,000 permutations) were calculated at the level of coastal Europe and at coastline or subbasin level (Baltic-Atlantic, Balearic, Tyrrhenian, and Adriatic-Ionian). To visualize genetic structure at subbasin or coastline level, a discriminant analysis of principal components (DAPC) was performed (Jombart et al., 2010) using Adegenet (Jombart, 2008) for R (version 3.3.3; R Core Development Team) with the number of principal components set to 20, following alpha-score indication. The number of clusters was set at *K* = 4. The probability of assignment for each individual was represented using the compoplot function of Adegenet. To test for significance of the observed global spatial pattern, a global Mantel test (Mantel, 1967) was performed for both pairwise population *F*_ST_ analog (Theta after Weir and Cockerham, 1984) and linearized [*F*_ST_/(1–*F*_ST_)]. Also, the slope [a regression over ln(distance)] over the full distance range was tested for Euclidean and for sea current distances (1-sided test, 1,000 randomizations).

#### Distance classes and permutation testing, breakpoint, and threshold analysis

Five distance classes as defined to obtain an equal number of pairwise comparisons of populations were considered in an ANOVA approach (1-sided test, 1,000 randomizations) for pairwise *F*_ST_ testing at four coastline regions (Baltic-Atlantic, Balearic, Tyrrhenian, Adriatic-Ionian). Similarly, an ANOVA approach was done for *R*_ST_ and allele permutation testing to verify for potential evolutionary signals over distance ranges within each subbasin. Based on the outcome of testing five distance classes, we additionally tested for isolation-by-distance (IBD) significance of the slope (regression over ln distance) over a relevant restricted distance range of 300 km (1-sided test, 1,000 randomizations), using the *F*_ST_ ANOVA approach for Euclidean distances. Euclidean distances and sea currents were equally representative over shorter distances within each subbasin.

A piecewise linear regression with breakpoint analysis was done, considering full distance ranges at the European level and at coastline level, using pairwise *F*_ST_ and geographic distance of populations. Additional data mining was done using a CRT analysis with *F*_ST_ as dependent variable and geographic distance as a continuous predictor. The tree structure with nodes, size of nodes, node mean, node variance, and a split variable determine a threshold value as estimate for connectivity within an IBD relevant context. Breakpoint and threshold analyses were performed with STATISTICA v.12.

To test for genetic IBD, an analysis of the pairwise kinship coefficients (*F*_IJ_) (Loiselle et al., 1995), namely a spatial autocorrelation at the most detailed geographic level, was done for the potentially relevant short distance classes (5, 20, 50, 100 km) and for the within-site class (1-sided test, 1,000 randomizations) of each subbasin, using SPAGeDi.

#### Ocean model and particle trajectories

We constructed a dispersal model to simulate dispersal trajectories and estimate dispersal distances. The model was forced using hourly ocean surface current data from a mesoscale and tide-resolving configuration of the Massachussetts Institute of Technology general circulation model (MITgcm; Hill et al., 2007). The MITgcm was initialized from a data-constrained global ocean solution provided by the ECCO2 project, i.e., Estimating the Circulation and Climate of the Ocean, Phase II (Menemenlis et al., 2008). Surface boundary conditions are from the 0.14° European Centre for Medium-Range Weather Forecasts (ECMWF) and bathymetry consists of a blend of the Smith and Sandwell (1997) v.14.1 and International Bathymetric Chart of the Arctic Ocean (IBCAO). Horizontal grid spacing of the ocean current data is *ca*. 4 km.

Propagules were released hourly during the period August 1 (00h00) to August 31 (23h00), 2012 and were advected by the simulated ocean surface currents for floating periods of 1, 2, 3, and 4 weeks respectively. End locations of the seeds for all four simulation periods are shown in **Figures 2A–D**. Since release locations consist of coastal locations, some may be situated on land in the model land-ocean mask, hampering propagation in the numerical simulations. To avoid this problem, the release locations were moved to the closest wet model grid cell, ensuring that virtual propagules can be transported by ocean currents. Dispersal distances were calculated using the spherical law of cosine and the longitude and latitude coordinates of the propagules' release and end locations (similar to the methodology of Van der Stocken et al., 2013).

#### Testing dispersal distances for genetic imprint variables

The obtained scenario of modeled dispersal distances and trajectories were compared as an overlay with spatial clustering using a spatial Bayesian method and a barrier detection method. Bayesian analysis of population structure (BAPS v. 6.0; Corander et al., 2003) partitions the data among groups of tetraploid individuals (i.e., detects which sampling locations are different) based on similarity of population allele frequencies, compared to a Fisher's exact test for population differentiation. Occurrence of many private alleles and strong differences in alleles will yield a high number of clusters, whereas comparable allele frequencies are expected to group into larger gene pools. The best partition of populations into K clusters was identified as the one with the highest marginal log-likelihood. The following settings were used: spatial genetic mixture analysis, clustering of individuals, K from 1 to 46 with 10 replicates. An admixture analysis was based on a mixture clustering of individuals (*K* = 4 and *K* = 11 were selected), with 10 replicates and *p* < 0.05 for the strain admixture (if not significant, then a single color bar was used for that individual). The minimum size of a population that will be taken into account for estimating admixture was set at 5 (50 iterations). An input number of 10 reference individuals from each population was considered (10 iterations). To check whether or not dispersal could be limited by physical barriers of opposite sea surface currents, rather than geographic distance, we calculated barriers to gene flow based on pairwise *F*_ST_ values at the level of each locus with BARRIER v. 2.2 (Manni et al., 2004). We used 10 cumulative matrices because concordance among 10 loci will reveal higher support for barriers (and *vice versa*, non-concordance will give high support for connectivity). The Monmonier maximum difference algorithm was run on the genetic distance matrices and geographical coordinates of sampling location to detect genetic breaks based on an *a priori* defined number of barriers. We followed an *a priori* assignment of three barriers, primarily testing for barriers between subbasins. The robustness of obtained major barriers was verified by running again with only one *a priori* barrier set, since the first boundary is the most important one.

## Results

### Basic population genetic variables

*Ruppia cirrhosa* populations can contain similar MLGs within a site, due to the mixed reproductive mode of clonal regrowth, in addition to a certain extent of unique genotypes resulting from sexual reproduction. The clonal richness (R) of 46 populations on average was 0.80 and ranging from *R* = 0.12–1 (Table 1). The fine-scale transects of only 1 m interval between ramets gave 36 out of 46 with *R* < 0.50. On average, populations from the Baltic-Atlantic had lowest clonal richness (0.64) whereas those from Balearic had highest (0.83). Similar MLGs were removed for all further analysis such that the number of ramets (*N* = 1303) was reduced to the number of genets (*G* = 955). Ten polymorphic loci revealed a total of 192 alleles in 46 *R. cirrhosa* populations across Europe.

Nine loci harbored 3 or 4 alleles within an individual under an autotetraploid condition, whereas one cross-amplified locus (*RUMR4*) showed maximum two alleles per locus. Those nine loci were the ones primarily designed from *R. cirrhosa RC3, RCS5, RCS8, RCS9, RCS27, RM3, RM12, RM26* and one cross-amplified locus from *R. maritima* (*RMB15*). These nine loci were not showing tetraploidy in concert but on average for only 1–3 loci at a time within an individual. At population level, one to eight loci showed more than 2 alleles in most *R. cirhosa* populations across Europe. Apparently, populations sampled along the North Sea (5_NL, 6_FR, 7_FR) and Atlantic coast (9_FR, 10_FR, 11_FR) had a maximum of 2 alleles for each locus, whereas only one small-sized water body in the Mediterranean contained maximum 2 alleles per locus (22_SP), potentially indicating a limited number of founders or multiple bottlenecks.

The mean number of alleles per locus in 955 individuals in European *R. cirrhosa* was 18.1 (Effective number *A*_e_ = 5.9) (Table 1). The Baltic-Atlantic populations had the lowest number of alleles (*A* = 9.1 and *A*_e_ = 3.4), whereas the Mediterranean populations had highest levels (*A* ranging between 10.4 and 13.4; *A*_e_ between 4.7 and 5.4; Table 1). Allelic richness on average in *R. cirrhosa* was 6.1 at k = 20. Gene diversities across Europe reached *H*_O_ = 0.533 and *H*_E_ = 0.744 (Table 1). However, much lower heterozygosity values were found in the Baltic-Atlantic (*H*_O_ = 0.435 and *H*_E_ = 0.624) than in the Mediterranean basins (*H*_O_ ranging from 0.556 to 0.573 and *H*_E_ from 0.704 to 0.743). The individual inbreeding *F*_i_ was significantly positive in 38 out of 46 populations, for each subbasin and for the total. Baltic-Atlantic populations showed the highest level of inbreeding (0.610), whereas Mediterranean subbasins had somewhat less elevated levels (0.480–0.526). Populations with lowest number of genets (5_GER, 6_NL, 19_SP, 32_IT, 33_IT, 40_GR and 42_GR) may show less reliable estimations of allele diversity, heterozygosities, or individual inbreeding.

Global ANOVA-based *F*-statistics (Table 2) were calculated at the level of Europe and at coastline region level (Baltic-Atlantic, Balearic, Tyrrhenian, Adriatic-Ionian), revealing at both hierarchical geographic levels a high value of overall inbreeding (*F*_IT_ = 0.574 ranging from 0.497 to 0.627), within-population inbreeding (*F*_IS_ = 0.361 ranging from 0.255 to 0.414) and populational differentiation (*F*_ST_ = 0.333 ranging from 0.228 to 0.365). Highest population differentiation was found along the Baltic-Atlantic coastline and weakest for the Balearic Sea (Table 2). An overall high *F*_IS_ referred to mating among relatives within a population (transects) because the estimated selfing rates were low and non-significant. *R*_IT_, *R*_IS_ and *R*_ST_ were nearly similar or lower than the *F*-based values, indicating no signal of global stronger differentiation due to allele size differences (not shown).

**Table 2:** AMOVA-based global *F*-statistics for 46 European *Ruppia cirrhosa* populations and four coastline regions.

	***N***	***F*_IT_**	***F*_IS_**	***F*_ST_**
Europe (total)	46	0.574	0.361	0.333
Baltic-Atlantic	10	0.627	0.414	0.365
Balearic Sea	15	0.534	0.396	0.228
Tyrrhenian Sea	7	0.510	0.255	0.342
Adriatic-Ionian Sea	14	0.497	0.297	0.284

*All F-values have p < 0.001*.

**Figure 1:**
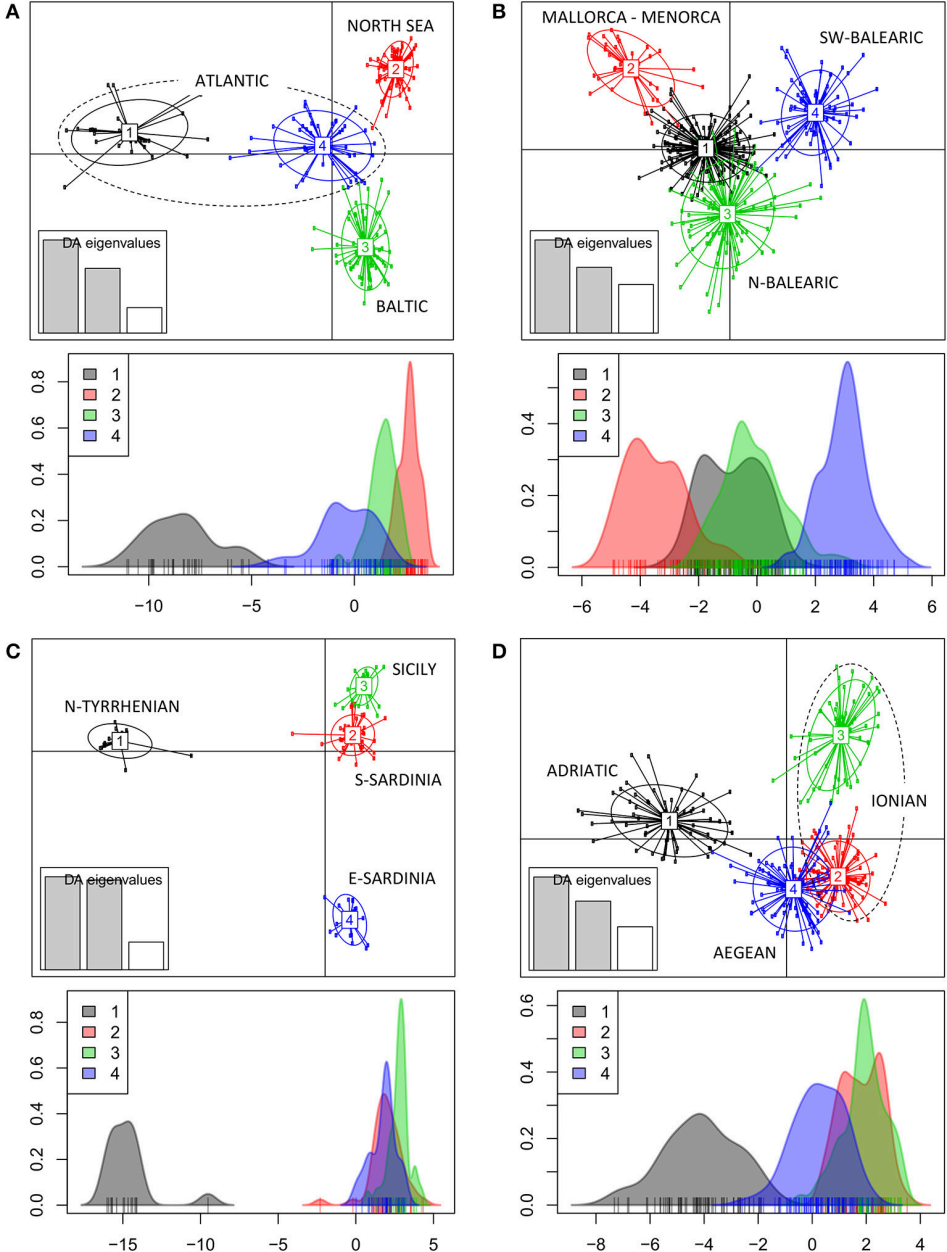
Clustering analysis of *Ruppia cirrhosa* populations within subbasins or coastlines. The DAPC was performed with original populations and colors represent the different clusters at *K* = 4 for each region. **(A)** Baltic-North Sea-Atlantic; **(B)** Balearic Sea; **(C)** Tyrrhenian Sea; **(D)** Adriatic-Ionian-Aegean Sea. Discriminant analyses of each corresponding data set are shown under each respective DAPC result, with x-axis showing the values of the first discriminant function and y-axis indicating the smoothed density of observations.

The DAPC of populations within each geographic region (Figure 1A) revealed a clear grouping of populations along a first and second axis according to regions of the Baltic (cluster 3), North Sea (cluster 2) and Atlantic coastline (clusters1 and 4). A second axis can be explained from an allele diversity gradient (namely, lowest *A*_e_ < 2 are in North Sea populations, whereas *A*_e_ > 2 was found in other sites, see Table 1). The DAPC of the Balearic Sea populations (Figure 1B) showed a strong mixing of individuals though separated along a first and second axis the island populations of Mallorca and Menorca (cluster 2) from the continental coastline populations along the N-Balearic (cluster 1 and 3) and the SW-Balearic (southern Iberian coastline, cluster 4). The DAPC of the Tyrrhenian Sea populations (Figure 1C) resulted in a very strong separation along a first axis of a population from the North Tyrrhenian coastline (cluster 1) and along a second axis of E-Sardinia (cluster 4) from a rather mixed group of S. Sardinia and Sicily (cluster 2 and 3). The DAPC of the East Mediterranean populations (Figure 1D) showed a clear grouping along the first axis of the Adriatic Sea (cluster 1) and a gradient along the second axis of Aegean and Ionian Sea populations (cluster 2, 3, and 4). Lake Prokopos along the Peloponnesos (cluster 3) was the most differentiated from the other Ionian lagoons.

### IBD and *F*_ST_ class permutation test over full distance range

All slopes of the regression with ln(distance) over the full range for all populations across Europe (2,518 km) and within each of the coastlines (563–1,497 km) were highly significant for either Euclidean distances or for sea current distances (Table 3), except for those of the Tyrrhenian Sea (not significant for Euclidean distances although marginally significant at *p* < 0.05 when considering sea current distances). The latter can be explained from the strongly differentiated populations on E. Sardinia. The slopes and *r*^2^ were higher for the populations of the Baltic-Atlantic (*r*^2^ = 0.30) and the Adriatic-Ionian (*r*^2^ = 0.57) than for Balearic and Tyrrhenian Sea (Table 3).

**Table 3:** Testing significance of IBD over full distance ranges across Europe and at coastline level.

	***N***	**km**	**b (slope)**	**a (intercept)**	***r*^2^**	***p***
**EUCLIDEAN DISTANCES**						
Europe	1,035	2,518	0.041	0.055	0.17	<**0.001**
Baltic-Atlantic	45	1,497	0.048	0.065	0.30	**0.002**
Balearic Sea	91	818	0.026	0.088	0.14	<**0.001**
Tyrrhenian Sea	21	563	0.022	0.220	0.07	ns
Adriatic-Ionian Sea	91	1,155	0.038	0.073	0.57	<**0.001**
**SEA CURRENT DISTANCES**						
Europe	1,035	8,194	0.036	0.056	0.18	<**0.001**
Baltic-Atlantic	45	2,684	0.041	0.085	0.28	**0.003**
Balearic Sea	91	1,630	0.029	0.059	0.12	**0.002**
Tyrrhenian Sea	21	1,190	0.025	0.192	0.12	**0.050**
Adriatic-Ionian Sea	91	2,280	0.038	0.065	0.56	<**0.001**

*F_ST_-ANOVA approach on Euclidean and sea current distances (1-sided test, H1: obs>exp) of regression with ln(distance). N, number of population pairs; km, maximum range; b, slope; a, intercept; r^2^, coefficient of determination and p-value = 1-sided test (H1: obs>exp). Significant values are in bold (ns, not significantly different from average)*.

At the level of four coastlines and subbasins (Baltic Atlantic, Balearic, Tyrrhenian, and Adriatic-Ionian), pairwise *F*_ST_ permutation testing of populations within 5 distance classes (Table 4) revealed significantly lower differentiation than average for the shortest distance classes, up to maximum 104 km and 128 km for Euclidean and sea currents respectively (Adriatic-Ionian), 198 km and 280 km (Balearic), and 152–154 km (Baltic-Atlantic), but not for populations in the Tyrrhenian Sea (first distance class of maximum 89 and 120 km respectively). Consistently higher *F*_ST_ values at larger distances (beyond 104 km) were found in the Adriatic-Ionian Sea. The average *F*_ST_ values within each subbasin were thus elevated, either already at very short distances (Tyrrhenian Sea) or beyond abovementioned maximum distances of 104-280 km, albeit short dispersal distances when considering the long coastlines and the size of the subbasins. However, the full-range distances are not fully explanatory because lower *F*_ST_ were observed in first distance classes but then suddenly increasing and already leveling off at high *F*_ST_ values at further distance classes (Table 4). A similar *R*_ST_ and allele permutation test was not relevant, indicating that allele length differences are not showing evolutionary signals within a subbasin and thus can be further treated as geographic entities (results not shown).

**Table 4:** Pairwise *F*_ST_ permutation testing (ANOVA approach) at 5 distance classes of *Ruppia cirrhosa* populations along four different coastlines using Euclidean and sea current distances.

**Euclidean distance classes**	**1**	**2**	**3**	**4**	**5**
**BALTIC-ATLANTIC**					
Number of pairs	9	9	9	9	9
Max distance (km)	154	687	841	1,186	1,497
Mean distance (km)	57	464	754	951	1,436
Pairwise *F*_ST_ Average = 0.348	0.167	0.378	0.445	0.404	0.346
P(1-sided test, H1: obs < exp)	<**0.001**	ns	ns	ns	ns
P(1-sided test, H1: obs>exp)	ns	ns	**0.030**	ns	ns
**BALEARIC SEA**					
Number of pairs	18	18	18	18	19
Max distance (km)	198	301	420	514	818
Mean distance (km)	99	254	380	469	644
Pairwise *F*_ST_ Average = 0.232	0.174	0.271	0.217	0.258	0.240
P(1-sided test, H1: obs < exp)	**0.002**	ns	ns	ns	ns
P(1-sided test, H1: obs>exp)	ns	ns	ns	ns	ns
**TYRRHENIAN SEA**					
Number of pairs	4	4	4	4	5
Max distance (km)	89	335	354	412	563
Mean distance (km)	46	292	340	397	493
Pairwise *F*_ST_ Average = 0.341	0.288	0.425	0.281	0.310	0.387
P(1-sided test, H1: obs < exp)	ns	ns	ns	ns	ns
P(1-sided test, H1: obs>exp)	ns	**0.019**	ns	ns	ns
**ADRIATIC-IONIAN SEA**					
Number of pairs	18	18	18	18	19
Max distance (km)	21	104	485	1,025	1,155
Mean distance (km)	11	75	238	856	1,064
Pairwise *F*_ST_ Average = 0.265	0.142	0.194	0.321	0.347	0.317
P(1-sided test, H1: obs < exp)	<**0.001**	**0.002**	ns	ns	ns
P(1-sided test, H1: obs>exp)	ns	ns	**0.015**	**0.003**	**0.026**
**SEA CURRENT DISTANCE CLASSES BALTIC-ATLANTIC**					
Number of pairs	9	9	9	10	8
Max distance (km)	152	1,240	1,427	2,532	2,684
Mean distance (km)	69	834	1,298	1,860	2,644
Pairwise *F*_ST_ Average = 0.348	0.164	0.449	0.369	0.419	0.330
P(1-sided test, H1: obs < exp)	<**0.001**	ns	ns	ns	ns
P(1-sided test, H1: obs>exp)	ns	**0.022**	ns	ns	ns
**BALEARIC SEA**					
Number of pairs	18	18	18	18	19
Max distance (km)	280	461	601	721	1,630
Mean distance (km)	139	388	533	670	1,094
Pairwise *F*_ST_ Average = 0.232	0.162	0.295	0.207	0.248	0.249
P(1-sided test, H1: obs < exp)	<**0.001**	ns	ns	ns	ns
P(1-sided test, H1: obs>exp)	ns	**0.008**	ns	ns	ns
**TYRRHENIAN SEA**					
Number of pairs	4	5	3	4	5
Max distance (km)	120	401	755	960	1,190
Mean distance (km)	70	370	599	904	1,142
Pairwise *F*_ST_ Average = 0.341	0.288	0.294	0.366	0.317	0.433
P(1-sided test, H1: obs < exp)	ns	ns	ns	ns	ns
P(1-sided test, H1: obs>exp)	ns	ns	ns	ns	ns
**ADRIATIC-IONIAN SEA**					
Number of pairs	18	18	18	18	19
Max distance (km)	22	128	1,047	1,141	2,280
Mean distance (km)	13	95	442	1,103	1,356
Pairwise *F*_ST_ Average = 0.265	0.142	0.202	0.333	0.321	0.323
P(1-sided test, H1: obs < exp)	<**0.001**	**0.004**	ns	ns	ns
P(1-sided test, H1: obs>exp)	ns	ns	**0.003**	**0.018**	**0.007**

*Significant values are in bold (ns, not significantly different from average)*.

### Connectivity at coastline or subbasin level using additional approaches

Because only 104–280 km appeared to be significant for connectivity when using the *F*_ST_ approach, additional IBD testing was done within a restricted range of 300 km. All distance classes beyond 300 km showed high *F*_ST_ and were not significantly lower than the average *F*_ST_. Hence, these could be deleted from further connectivity estimations. A regression of the population differentiation over a geographical range restricted to 300 km [as ln(km) in Table 5] revealed an overall better *r*^2^ than previous testing over a full distance range (Table 3) and gave a highly significant IBD across Europe and within most subbasins with a comparable slope (b) though a smaller intercept (a). Too few population pairs (only five) at <300 km were available for the Tyrrhenian Sea to adopt this approach of restricted regression, though the intercept was also high (*a* = 0.2), indicating strong differentiation over shortest distances.

**Table 5:** Testing for significance of IBD slopes [regression with ln (distance)] up to 300 km Euclidean distance range at European and at coastline level using the pairwise *F*_ST_ ANOVA approach.

	***N* pairs**	**Range**	**b**	**a**	***r*^2^**	***p***
	**pairs**	**km**	**(slope)**	**(intercept)**		
Europe	83	300	0.039	0.061	0.26	<**0.001**
Baltic-Atlantic	12	300	0.069	0.017	0.30	**0.009**
Balearic Sea	18	300	0.028	0.040	0.30	**0.020**
Tyrrhenian Sea	5	300	0.027	0.200	0.34	ns
Adriatic-Ionian Sea	48	300	0.043	0.050	0.43	<**0.001**

*N, number of population pairs; b, slope; a, intercept; r^2^, coefficient of determination and p-value = 1-sided test (H1: obs>exp). Significant values are in bold (ns, not significantly different from average)*.

When considering the full distance ranges at European and subbasin level in a piecewise linear regression (i.e., a breakpoint analysis) of the pairwise population differentiation (*F*_ST_), the *r*^2^ (ranging from 0.56 to 0.90) were substantially improved over the previous analyses with single regression because IBD appears most significant over shorter geographic distances (Table 6). Due to the flat slope of the second regression line, covering the large distance values, the breakpoints (i.e., the crossing of two regression lines) were obtained at larger geographic distances than expected from previous analysis over restricted distances. Breakpoints were at 1,187 km (*F*_ST_ = 0.317) for *R. cirrhosa* in Europe, 954 km (*F*_ST_ = 0.335) for Baltic-Atlantic, 357 km (*F*_ST_ = 0.213) for Balearic Sea, 322 (*F*_ST_ = 0.341) for Tyrrhenian Sea and 507 km (*F*_ST_ = 0.263) for Adriatic-Ionian Sea. Piecewise linear regression yielded a much better explained variance, the obtained breakpoints were shifted toward the longer geographic distances.

**Table 6:** Breakpoint analysis of two IBD regressions considering full distance ranges at European and at subbasin level using the pairwise *F*_ST_ ANOVA approach.

	***N* pairs**	***r*^2^**	***F*_ST_**	***r*^2^**	**km**
Europe	1,035	0.68	0.317	0.74	1187
Baltic-Atlantic	36	0.8	0.335	0.76	954
Balearic Sea	78	0.56	0.213	0.72	357
Tyrrhenian Sea	21	0.77	0.341	0.82	322
Adriatic-Ionian Sea	66	0.73	0.263	0.9	507

*N, number of pairs; r^2^, coefficient of determination for each regression; breakpoint value for F_ST_ and corresponding geographic distance (km)*.

A CRT analysis at European and subbasin level (Table 7), with *F*_ST_ as dependent variable and the full geographic distance range as a continuous predictor, revealed a tree structure with first nodes at low variance. This yielded a threshold value of 836 km (node 1, at *F*_ST_ = 0.317) and of 88 km (node 2, at *F*_ST_ = 0.262) at the Europe level, 179 km (Baltic-Atlantic at *F*_ST_ = 0.335), 78 km (Balearic Sea at *F*_ST_ = 0.213), 47 km (Tyrrhenian Sea at *F*_ST_ = 0.321 *for node 2 because a less relevant node 1 only had split 2 cases against 19 at km 514 and F*_ST_ *0.341*) and 108 km (Adriatic-Ionian Sea at *F*_ST_ = 0.263). This estimate of thresholds to connectivity within each coastline or subbasin thus indicates short distances of 47–179 km (about half the value as obtained from an ANOVA approach: 104–280 km, see Table 4), and appears to be a relevant approach to estimate thresholds within an IBD context.

**Table 7:** Classification and regression tree analysis.

	**Node**	***N* cases**	**Mean *F*_ST_**	**Variance**	**Split km**
Europe	1	1,035	0.317	0.013	836
	2	352	0.262	0.013	88
	3	683	0.345	0.011	1972
Baltic-Atlantic	1	36	0.335	0.024	179
	2	5	0.100	0.005	0
	3	31	0.373	0.016	833
Balearic Sea	1	78	0.213	0.008	78
	2	8	0.099	0.007	9
	3	70	0.226	0.006	303
Tyrrhenian Sea	1	21	0.341	0.014	514
	2	19	0.321	0.011	47
	4	2	0.206	0.001	0
Adriatic-Ionian Sea	1	66	0.263	0.009	108
	2	24	0.162	0.005	3
	3	42	0.320	0.003	1050

*Threshold values of geographic distance (Split km) in relation to mean F_ST_ values at three nodes for number of cases, mean F_ST_, F_ST_ variance and threshold value at split of geographic distance (km)*.

The above mentioned pattern of distance-related differentiation of populations (“ecological IBD”) was further elaborated at the individual level (“genetic IBD”) using kinship coefficients at 4 distance classes that were hypothesized to be relevant for contemporary connectivity (5–20–50–100 km). The within-population kinship coefficient for *R. cirrhosa* reached *F*_IJ_ = 0.28 along the Baltic-Atlantic coastline, 0.24 in the Tyrrhenian Sea, 0.21 in the Adriatic-Ionian Sea and was lowest at 0.16 for the Balearic Sea (Table 8). All obtained values should be considered high (an *F*_IJ_ = 0.25 corresponds to full-sibling mating) and reflect their individual relatedness within the sampled transects of 30 m length each. As expected, the individual kinship declines with geographic distance but showed high *F*_IJ_ = 0.10–0.20 within 5 km (these were adjacent sites) and in several cases, even up to 20 km (these were large and open lagoons with repeated transect sampling) but not beyond that range (Table 8). This “genetic IBD” analysis indicates that spatial autocorrelation can be relevant within distances up to 20 km, thereby demonstrating a contemporary and detectable connectivity.

**Table 8:** Pairwise kinship coefficients (*F*_IJ_) of *Ruppia cirrhosa* individuals at shortest distance classes of neighboring lagoon populations within each coastal basin.

**Coastline**	**Intra-pop**	**5 km adjacent**	**20 km open**	**50 km among**	**100 km among**
**distance classes**	**transect 30 m**	**sites**	**lagoon area**	**lagoons**	**lagoons**
**BALTIC-ATLANTIC (205 INDIVIDUALS)**					
Number of pairs	1,828	2,239	1,371	0	520
Mean distance (km)	–	2.1	8.7	–	96
Pairwise kinship coefficients	**0.275**	**0.204**	**0.244**	–	−0.027
P(1-sided test, H1: obs>exp)	<**0.001**	<**0.001**	**0.003**	–	ns
**BALEARIC SEA (337 INDIVIDUALS)**					
Number of pairs	4,033	2,081	812	1,344	2,233
Mean distance	–	2.8	16.7	23	78
Pairwise Kinship coefficients	**0.164**	**0.102**	**0.097**	0.029	0.066
P(1-sided test, H1: obs>exp)	<**0.001**	<**0.001**	**0.042**	ns	ns
**TYRRHENIAN SEA (114 INDIVIDUALS)**					
Number of pairs	905	186	0	432	817
Mean distance	–	0.7	–	35	77
Pairwise Kinship coefficients	**0.244**	**0.152**	–	−0.011	−0.089
P(1-sided test, H1: obs>exp)	<**0.001**	<**0.001**	–	ns	ns
**ADRIATIC-IONIAN SEA (280 INDIVIDUALS)**					
Number of pairs	2,941	2,716	3,505	3,102	1,496
Mean distance	–	2.0	15	28	90
Pairwise Kinship coefficients	**0.211**	**0.171**	**0.045**	0.010	0.064
P(1-sided test, H1: obs>exp)	<**0.001**	<**0.001**	**0.050**	ns	0.012

*Significant values (in bold) were found at < 20 km distance*.

**Figure 2:**
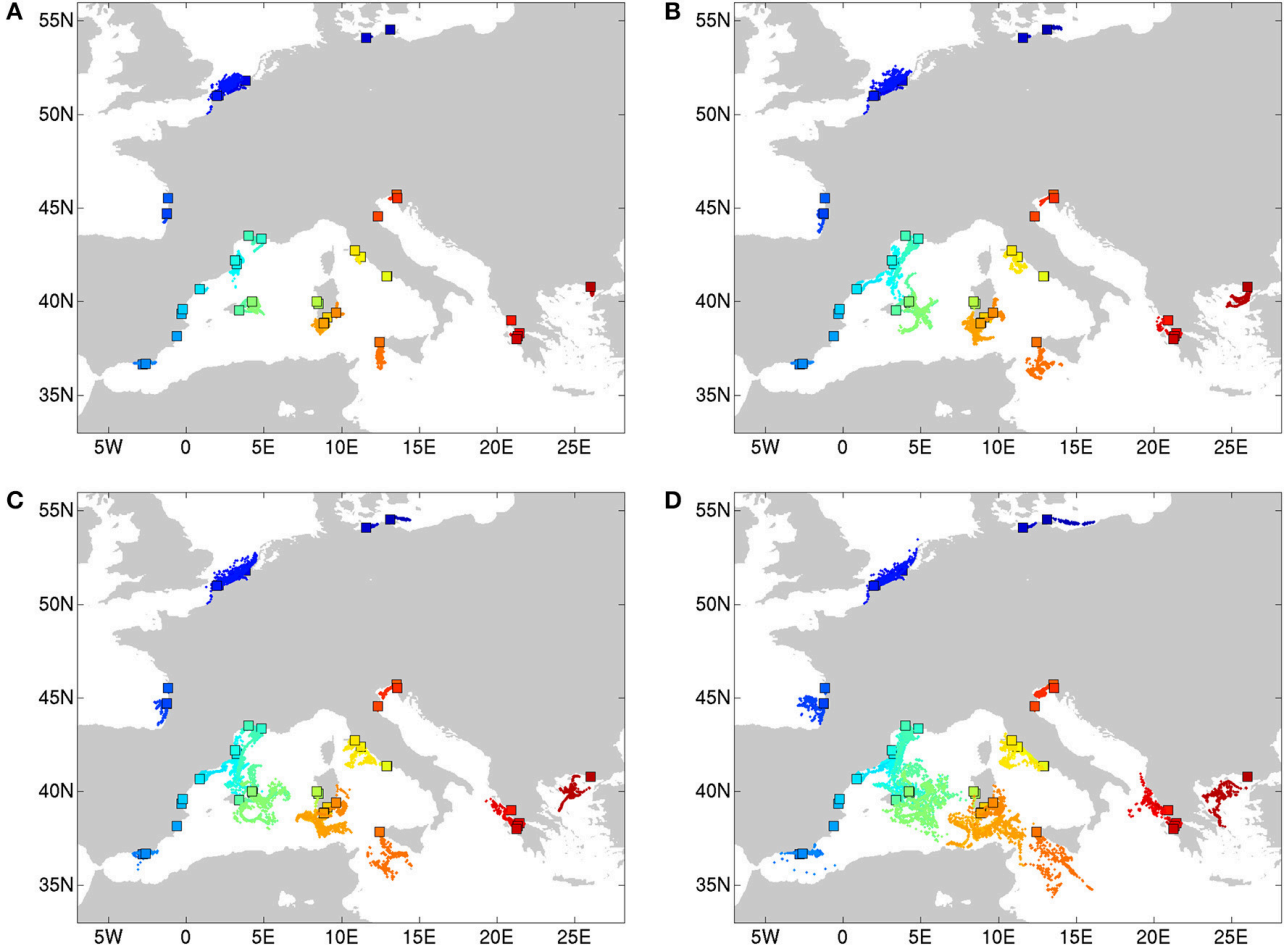
Simulated end locations of particles (seeds) released in different locations (rectangles, color code) along the Mediterranean (Balearic, Tyrrhenian, Ionian), and the Atlantic to Baltic Sea coastline. Particles were released hourly over the period of 1 August (00h00) 2012 to 31 August (23h00) 2012, and were transported passively by simulated ocean currents. End locations (dots) were simulated for seeds with floating periods of 1 week **(A)**, 2 weeks **(B)**, 3 weeks **(C)**, and 4 weeks **(D)**.

Simulated end locations of particles released along coastal Europe show a clear increase in dispersal distances with increasing propagule (i.e., floating shoots bearing fruits) floating periods. Particles with a floating period of 1 week (Figure 2A) do not allow particles to cover vast distances during the release period considered, with propagules remaining rather close to their release location. For floating periods of 2 weeks and more, propagules from certain locations are able to reach more remote localities. This is especially true for dispersal in the Balearic and the Tyrrhenian Sea, in contrast to much shorter dispersal along the other coastlines and in other subbasins. The spatial spread of end locations for propagules from the same release location (color code) reflect the sensitivity of dispersal trajectories to temporal variability in ocean current strength and direction, and as such also the timing of seed release.

Median dispersal distances of outgoing propagules from sites were simulated and reached between 4.4 and 43.8 (sd 15.9) km for Baltic—North Sea—Atlantic; between 2.2 and 200.1 (sd 81) km for the Balearic, between 4.4 and 221.3 (sd 83.6) km for the Tyrrhenian and between 3.1 and 194.6 (sd 69.1) km for the Adriatic-Ionian-Aegean subbasins.

**Figure 3:**
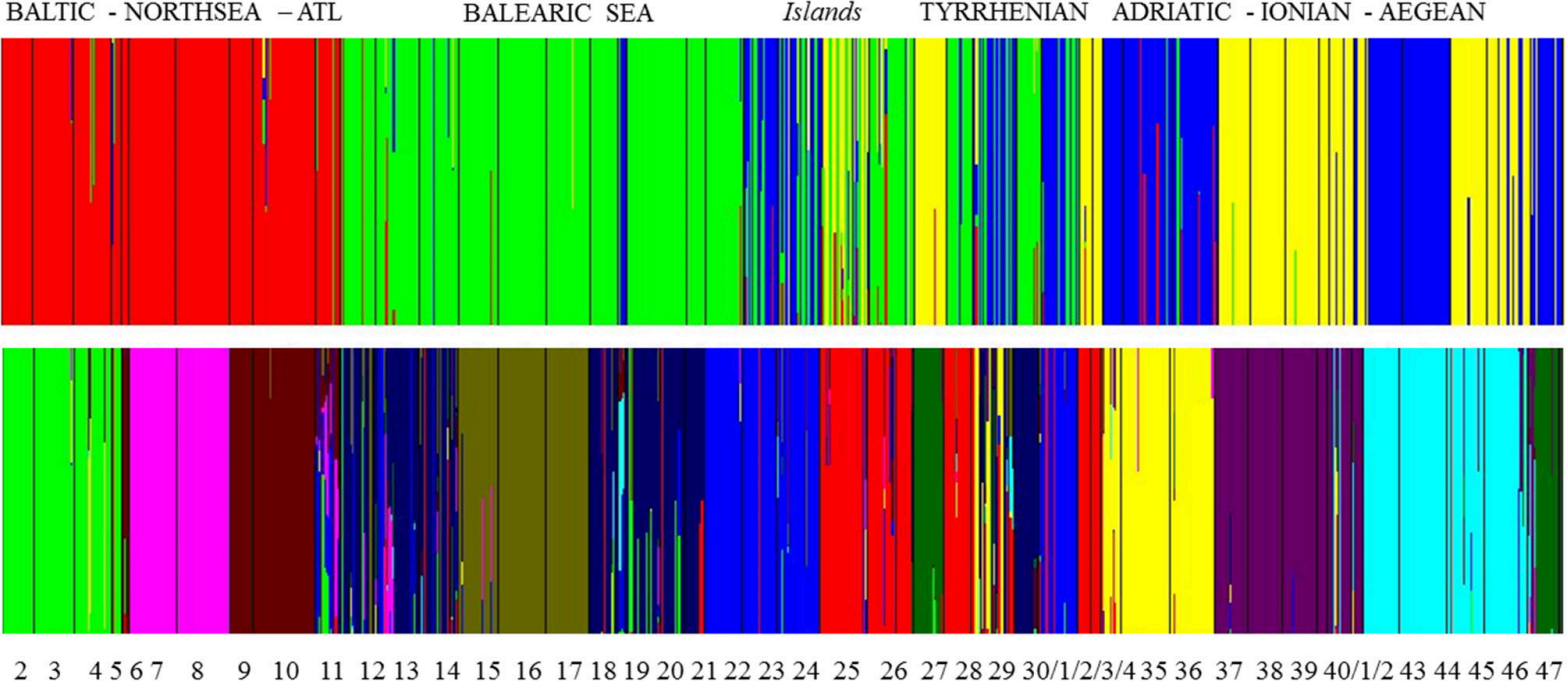
Bayesian analysis of population structure (BAPS) at *K* = 4 and at *K* = 11 gene pool clusters showing admixed individuals (i.e., vertical bars—on a scale from 0 to 100% —of individuals assigned to more than one gene pool) within clusters and mostly encountered for Balearic Sea, Islands, and Tyrrhenian Sea.

A group level mixture analysis (BAPS) of individual multilocus genotypes from 46 populations revealed an optimal partition for *K* = 34 clusters [Log (marginal likelihood) = −30 424 and probability of 0.44] or *K* = 35 clusters [Log (ml) = −30 424 and probability of 0.45]. These clusters correspond to a lagoon level and grouped only the open, connected sites within close vicinity (Table S2). An individual level mixture analysis was run for various *K* values ranging from *K* = 2 [Log (ml) = −36 858] to *K* = 15 [Log (ml) = −49 122]. To test for an eventual strong structure at a wide geographic level (Figure 3), *K* = 4 was tested [Log (ml) = −45 264] and gave a clear separation of the homogeneous (namely limited number of admixed individuals) cluster of Baltic—North Sea—Atlantic populations. Within the Mediterranean, there was a West-East gradient with very clear assignment of populations from the Balearic, though with admixtures, especially in the Balearic Islands and W. Sardinia. Populations from Sardinia and the Tyrrhenian Sea contained individuals that could be assigned to three different clusters whereas populations from the Adriatic-Ionian-Aegean clustered as entities with limited cases of admixtures. Thus, at *K* = 4 only the Baltic-North Sea-Atlantic was assigned as a single gene pool, fully separated from the Mediterranean.

**Figure 4:**
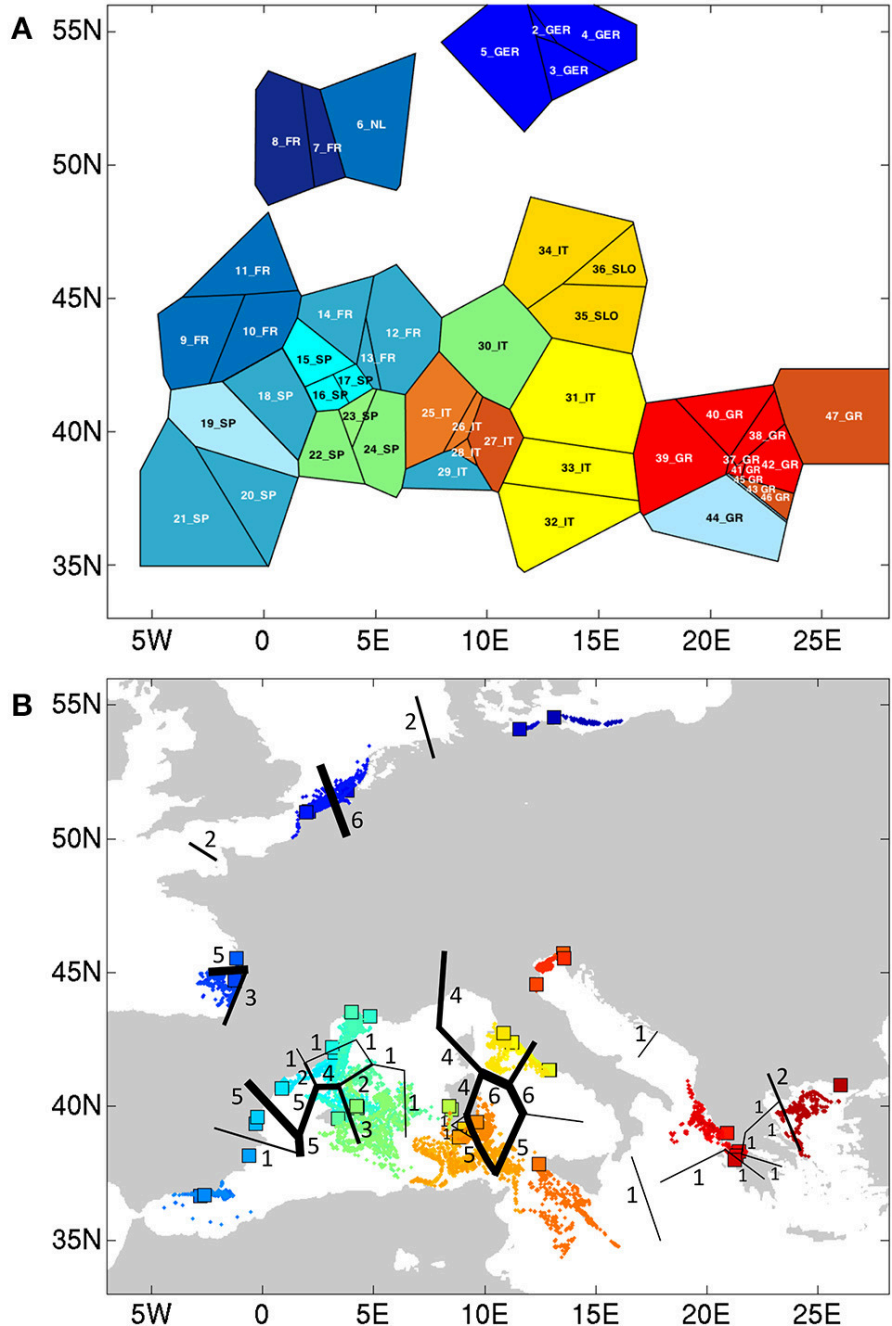
**(A)** Spatial BAPS (*K* = 11) with assignment of populations as a group, and **(B)** BARRIER detection with indication of genetic breaks (numbers of loci showing the breaks).

At *K* = 11 [Log (ml) = −38 590] an optimal resolution was obtained for each of the coastlines or subbasins (Figure 3), indicating a strong differentiation, that is congruent with the DAPC for each subbasin or coastline (Figure 1). Groups of individuals remained very consistent with entities that are geographically close. Remarkably, several populations (11_FR) and major areas along the Balearic coastline (Rhône Delta, 12_13_14_FR; Elbe Delta, 18_SP) appear to be very mixed without clear assignment to any of the clusters. Mixing of individuals is largest in the Balearic and the Tyrrhenian Sea but remained limited in the other coastlines or subbasins. This corresponds with modeled dispersal trajectories in both basins, where overlapping end locations of simulated dispersal trajectories suggest potential mixing (Figure 2). Even so, a few distant populations were assigned to a same gene pool, namely 25_26_IT (W. Sardinia), 28_IT (S. Sardinia) and 32_33_IT (Sicily) and corresponded to long and overlapping dispersal trajectories (see Figure 2). A spatial clustering at group level (*K* = 11) assigned populations (Figure 4A) according to either a geographic entity or according to an admixture of individual admixtures as noticed in the previous analysis (Figure 3). The latter admixtures blur the potential relationship to connectivity but again emphasized the strong differentiation between and within most coastlines and subbasins. A BARRIER analysis revealed regions with strong differentiation between neighboring populations (Figure 4B). Out of 10 loci, about half (4–6 loci) contributed to the barrier indication for populations, clearly forming a genetic break along coastlines of the North Sea (6_NL vs. 7_8_FR); SW Atlantic (9_10_FR vs. 11_FR); Balearic (19SP vs. 20_SP vs. other neighbors); and Tyrrhenian (27_IT vs. others; 28_IT vs. others). Only limited support for barriers, as indicated by two loci only, were revealed between the Baltic vs. North Sea, North Sea vs. Atlantic, NE Spain vs. Menorca (Balearic Island), and Aegean vs. Ionian coastlines.

## Discussion

We tested for an *a priori* microsatellite based population structure considering the assumption of a pre- and post LGM colonization genetic imprint (Triest and Sierens, 2014) and considering contemporary sea currents, using a suite of approaches. In traditional geostatistical studies, it is usually recommended to sample the whole study area but in such a way that various scales are investigated, containing both close and distant pairs of sampling sites (Diggle and Lophave, 2006; Diggle and Ribeiro, 2007). When investigating genetic patterns, quality depends more on a well-designed sampling strategy than on the detailed differences of a genetic marker system (Meirmans, 2015). We especially focused on a large dataset covering coastal populations of *R. cirrhosa* along major European coastlines and islands, including within-area comparisons for large lagoon systems.

Any dataset of alleles, loci, individual genotypes, and populations within a spatial context has a multivariate nature. Therefore, one might expect different levels of organization to be detected from genetic structure analysis over a continent-wide geographical scale. Our results on *R. cirrhosa* showed a congruence between genetic differentiation (barriers), genetic structure, threshold distances of connectivity and hydrodynamics (dispersal trajectories), and therefore rejected the null hypothesis of a completely random process of showing no structure. In *R. cirrhosa*, a complex IBD pattern with genetic breaks at European and at subbasin level was obtained including some mixture of populations as a group though with admixed individuals. When a study area is homogenous in terms of gene flow patterns and dispersal, a Mantel test makes sense. It is thus possible to get further insights into details of genetic variation by computing these statistics locally (Guillot et al., 2009). This was done for *R. cirrhosa* when testing for a historical “ecological” IBD within each subbasin using a restricted regression over shorter distances and when testing a contemporary “genetic” IBD over several km in the same lagoons (*F*_IJ_). Decomposition of genetic variance is a better method than a Mantel test (Wang et al., 2012; Hernawan et al., 2017). We proposed an alternative approach for decomposition of genetic variance and used a combination of Classification and Regression trees, dispersal trajectory modeling, Bayesian gene pool clustering, and Barrier detection. Overall, major patterns of connectivity could be obtained from the genetic imprint.

### Local contemporary dispersal and inferred connectivity range

Movement of genes in *R. cirrhosa* can only be considered as being realized when epihydrophylous pollination, seed formation, seed dispersal, germination and seedling establishment are successful. Seagrass populations may form at first by initial seedling recruitment (Eriksson, 1993), followed by local vegetative expansion and further local sexual recruitment, leaving the possibility of recurring seed dispersal into established populations (Becheler et al., 2014). An overview and gradient of reproductive characteristics of seagrasses (Kendrick et al., 2012) placed *Ruppia* among the genera with high seed density and a so-called small seed shadow. However, a high seed density is not necessarily the case for most marine populations of *R. cirrhosa* in Europe as this species had the lowest amount of seeds when compared to continental, inland European *R. maritima* and *R. drepanensis* (Triest and Sierens, 2013). *Ruppia cirrhosa* rather resembles *Zostera* spp. in terms of estimated movement paths (cf., conceptual Figure 2 in McMahon et al., 2014) over space (ca. 1–100 km) and time (days to weeks). The greatest opportunity for LDD movement and demographic connectivity among distant populations within contemporary time frames is through seeds, whereas over smaller spatial scales, the movement of pollen is imperative for maximizing cross-pollination (McMahon et al., 2014). In *R. cirrhosa* cross-pollination is promoted through release of pollen on the water surface. However, it can be subject to mating between relatives and cause (biparental) inbreeding within patches of a lagoon. Generally, local *R. cirrhosa* populations seem to be maintained by high genet turnover and by frequent seedling recruitment besides vegetative regrowth from persistent rhizomes. A local seed bank may stabilize after yearly replenishment and recruitment originated mainly within the same lagoon. In *Zostera noltii*, the absence of recent 1st and 2nd generation migrants indicated that LDD is rare although migrants may have entered during the life-span of a meadow (Jahnke et al., 2016). Despite a generally high genotypic diversity and allelic richness, we detected signs of inbred individuals at nearly all sites, thereby indicating a very high rate of local pollination. Pollination is supposed to occur within close vicinity. *Ruppia cirrhosa* is clearly outcrossing and therefore biparental inbreeding is the most likely explanation for the obtained levels of inbreeding. The overall *F*_IS_ values of 0.36 were much lower than in the truly and predominant selfing *R. maritima* (Triest and Sierens, 2015).

Observations of high genetic diversity levels in many *R. cirrhosa* meadows and a weak spatial structuring among meadows within a large or connected lagoon area (5–20 km) suggest that sexual reproduction is more important than previously thought. Overall, dispersal in seagrasses is far more likely to occur through seeds than through pollen (Kendrick et al., 2012), whereas meadows are not always dominated by clonal processes of regrowth or vegetative fragment dispersal. Seagrasses (also *Ruppia*) invest significant amounts of energy in sexual reproduction. In *R. cirrhosa* there is a high allelic and gene diversity but also limited clonal regrowth. High levels of local dispersal are expected from within-lagoon processes such as water currents, wind action and water birds). The lagoon inhabiting *R. cirrhosa* bears less fruits than e.g., *R. maritima* but the former can disperse as drifting detached shoots with fruit-developing inflorescences. Rafting is frequent within lagoons as can be observed from piled up mats at the edges. Rafting might occasionally occur along coastal stretches, given that the lagoons are open to the sea. In *Zostera marina*, reproductive floating shoots gave a high variance of inferred or simulated dispersal distances, ranging from 0.7 to 150 km (Orth et al., 1994; Harwell and Orth, 2002). Such data are lacking for *Ruppia*. Despite mature achenes are not buoyant when dehisced, their shoots bearing ripening inflorescences definitely can float at least for about 10 km (pers observ. L. Triest, August 2008) when washed outside the Arcachon lagoon (France-Atlantic coast) and stranded along the shore of the main coastline.

The local dispersal and contemporary connectivity was visualized by the Bayesian analysis that defined a very high number of gene pools (*K* = 35 as least negative marginal likelihood and highest probability) and by a corresponding fine-scaled genetic structure with significant *F*_IJ_ up to distance classes of 20 km.

This can be understood from a Bayesian analysis that defined groups of populations that are maximally differentiated from each other. In *R. cirrhosa*, we found the clustered populations to reflect the highest level of contemporary connectivity within the large open coastal lagoons, e.g., Hiddensee (2_GER, 3_GER, 4_GER); Arta lagoon (37_GR, 38_GR, 39_GR, 40_GR), amongst others. BAPS determines the number of genetically distinct populations, thereby assuming Hardy-Weinberg and linkage equilibrium, as well as a low mutation rate (Corander et al., 2003) that should be comparable to the kinship approach over relevant short distances within reach of single propagules. When compared to STRUCTURE, BAPS differs in treating populations from prior user-defined spatial information and in calculating allele frequencies in re-defined populations, which represents a more sophisticated estimator of basic allele frequency differentiation among populations (Pearse and Crandall, 2004).

Dispersal trajectories and estimated dispersal distances confirmed abovementioned observations, with median dispersal distances ranging between 4.4 and 43.8 (sd 15.9) km for the Baltic Sea-North Sea-Atlantic, between 2.2 and 200.1 (sd 81) km for the Balearic Sea, between 4.4 and 221.3 (sd 83.6) km for the Tyrrhenian Sea, and between 3.1 and 194.6 (sd 69.1) km for the Adriatic-Ionian-Aegean subbasins. Maximum dispersal distances after 4 weeks ranged between 3 km (e.g., Oristano, 26_IT) up to more than 400 km (e.g., populations of Carnon and Camargue, 12-14_FR), while minimum dispersal distances ranged between zero (no movement) to 64 km (Monolimni, 47_GR). Interestingly, releases in the population of Nord-Pas-de-Calais (7-8_FR) show pronounced dispersal distances already after 1 week of coastal transport (Figure 2A). Vast dispersal ranges (exceeding 300 km) can also be noted for populations of Sardinia and Sicily and populations in the Aegean-Ionian Sea, for floating periods of 1 month (Figure 2D). Most end locations, however, are located in the open ocean (Figure 2D), while the potential for establishment following stranding depends on habitat suitability.

Hydrodynamic modeling was used to infer connectivity between populations of a brown algae (*Cystoseira amentacea* in Marseille Bay) and to test the correlation between genetic and hydrodynamic connectivity using a model simultaneously considering local winds, river inputs and offshore circulation. A strong genetic structure however was not correlated with geographical distance (thus a lack of IBD), but zygotes may travel 4–18 h over distances up to 23 km (Thibaut et al., 2016). Inferred connectivity in *R. cirrhosa* thus was up to a similar distance of about 20 km which can be regarded as a relevant distance for rafting of detached fruit-bearing shoots.

### Historical connectivity and genetic breaks within subbasins

Beyond the abovementioned distance class of 20 km that appears to reflect contemporary connectivity in hydrologically connected sites, a CRT analysis delivered threshold values for accumulated historical dispersal events and indicated connectivity distances up to 47–179 km, depending on the empirical data from each subbasin or coastline. A breakpoint and threshold analysis resulted in very similar *F*_ST_ thresholds but gave very different geographic distances to infer connectivity. The obtained breakpoints were not accurate and too much influenced by high differentiation at large distances. A breakpoint analysis therefore is not an appropriate method, despite its much better explained variance. From our results, we argue that more accurate detection of genetic breaks can be estimated from CRT and additionally inferred from spatial BAPS and barrier detection.

BAPS estimates for admixed individuals within groups ranged from *K* = 4 to 15 (we presented *K* = 4 and *K* = 11), showing either clear geographic entities (i.e., Baltic—Atlantic) or a mixing of gene pools, especially of Balearic and Tyrrhenian Sea populations. Importantly, the number of *a priori* defined genetic groups is required (Guillot et al., 2009). Uncertainty in the estimation of a single *K* might just reflect different demographic processes (Meirmans, 2015). Therefore, in *R. cirrhosa*, we first tested many clusters in BAPS, verified the admixed situations and used BARRIER as a second independent approach. Barrier detection using the Monmonnier's algorithm (Monmonnier, 1973) is an approach that tries to identify pairs of neighboring pre-defined population units that display relatively large genetic differentiation. Accuracy improves when the number of matrices increases. Therefore in *R. cirrhosa*, we used distance matrices of each locus instead of a global pairwise *F*_ST_ only.

Differentiation was high within subbasins (*F*_ST_ = 0.23–0.37) and genetic breaks were empirically estimated at 104–280 km from a distance class permutation test. Consequently, because of these genetic breaks, we did not find good and clear evidence for IBD using simple regression over longer distances. *Zostera noltii* of Black Sea populations had very high pairwise *F*_ST_ values reaching values of 0.54 for an average reciprocal distance between sites of 360 km (Jahnke et al., 2016). Genetic breaks were found at ca. 65–150 km in *Zostera noltii* (Chust et al., 2013). These inferred distances are thus of a same magnitude for *Ruppia* and *Zostera*, although *Zostera* is growing more seaward.

Correlation coefficients between geographic and genetic distances (or the parameters of a variogram in spatial autocorrelation) are global statistics in the sense that their computation involves “all” data points and they reflect global properties of the populations sampled over the whole study area. IBD is often significant but could be an artifact of sampling design (Becheler et al., 2014). However, if *F*_ST_ differentiation is also the result of drift or natural selection, then the slope of this regression cannot be used to infer migration or dispersal distances (Holsinger and Weir, 2009) as it masks potential thresholds of realized connectivity. Significant IBD commonly results from discontinuities rather than continuous increase in *F*_ST_ with linear distance whereby only few geographically distant population pairs are causing a significant relationship (e.g., Muñiz-Salazar et al., 2006; Oetjen and Reusch, 2007; Tanaka et al., 2011; Sinclair et al., 2014).

The *r*-values make sense only when the study area is homogenous in terms of gene flow patterns and dispersal. When considering a “genetic IBD” (Malécot, 1951) instead of “ecological IBD” (Wright, 1943; Ishida, 2009), then an empirical spatial autocorrelation function brings insight into the spatial scale of variation of the contemporary process, in particular a characteristic distance at which statistical dependence disappears (called “range” in geostatistics). Therefore, spatial autocorrelation at restricted ranges over the shortest distance classes using *F*_IJ_, provides an alternative outcome. Additionally, clustering methods (Bayesian methods e.g., BAPS; Corander et al., 2003) look for homogeneous spatial domains of grouped populations and admixture of individuals in populations, whereas barrier detection methods (e.g., BARRIER, Manni et al., 2004) try to identify areas of abrupt discontinuities. As such, clustering and barrier methods are complementary (Guillot et al., 2009). Restricted dispersal, leading to *F*_ST_ differentiation over shorter distances, can be studied by IBD models, whereas differentiation induced by landscape or seascape barriers to gene flow is achieved from clustering models. Restricted dispersal distance and barriers are two factors that interplay. One factor (e.g., barrier effect) can be confounding to assess the other factor, e.g., dispersal distance from a smooth IBD (Guillot et al., 2009). Most quantitative approaches thus are based on idealized and abstract models that leave limited possibilities of associations with biological relevant parameters of populations, experiencing bottlenecks and multiple founders of different origins. Coastal, marine and freshwater species are studied with the same statistical tools as for terrestrial species, despite the obvious differences in terms of habitat features and dispersal processes. Incorporation of ecological and oceanographic information into a seascape genetics study was regularly proposed (Galindo et al., 2006; Kalinowski et al., 2008; Selkoe et al., 2008; Kendrick et al., 2017), but there is a need for more realistic models that include species and context-specific knowledge. The overall genetic structure appeared to be very congruent with subbasins and coastlines (*K* = 11) using BAPS, BARRIER, and a dispersal model that was forced using high-resolution oceanographic data as an overlay at population level. Dispersal trajectories remained mostly along the same coastline (NW Balearic Sea, Adriatic Sea, Ionian Sea, Baltic Sea). Genetic breaks can be matched with these routes because a portion of propagules remains locally, while an important part is transported off-coast with positions in the open ocean after 4 weeks of floating. Longer floating periods could allow for sporadic stranding on other coastlines, and eventually result in connectivity if establishment conditions are suitable.

Signals of admixture were observed in the Balearic and Tyrrhenian Sea populations. This may be due to the larger connectivity during and after the LGM (Triest and Sierens, 2014). Admixture can result from multiple founders or from hybridization that is known to occur frequently in *Ruppia* (Triest and Sierens, 2010; Ito et al., 2013; Martinez-Garrido et al., 2016). In our approach, we however focused on formerly confirmed pure *R. cirrhosa* and did not include the previously reported hybrid populations (Triest and Sierens, 2014, 2015), though one should not exclude remnant influences of introgressive hybridization.

One should bear in mind that the actual dynamics of the Atlantic coastline and Baltic Sea do not anymore reflect the potential of post-LGM colonization events. Therefore, the resulting genetic structure is supposed to be less related to contemporary dispersal routes along the Western European coastline. When IBD does not explain the genetic structure, then hydrodynamics and trajectory modeling might be relevant to investigate the presence of potential genetic breaks. The absence of clear IBD can be explained from the presence of distinct genetic clusters that break the IBD pattern. Such genetic structure could reflect ancient genetic patterns at a time when sea level was far below its present level, and the shape of the coastline and geographical barriers was different from the current one (e.g., Mediterannean basin = historical, Pleistocene /LGM; Atlantic-Baltic = post-LGM). Indications of possible LDD has been rarely assessed using both genetic assessments and physical modeling of ocean currents (Jahnke et al., 2016). A strong regional differentiation also was consistent with long-term barriers to dispersal persisting in marine environment through many sea level fluctuations, for *Posidonia australis* in SW Australia based on a hydrodynamic model (Sinclair et al., 2016). Contemporary genetic connectivity of *P. australis* was supposed to maintain through seed dispersal. Recurrent lack of IBD has been suggested to result from stochastic large-scale dispersal events (Kendrick et al., 2012) or from ancient polymorphisms of vicariance (Arnaud-Haond et al., 2014). *F*_ST_ integrates over a long time (thousands of generations) and is responding slowly to contemporary changes in connectivity (Lloyd et al., 2013), thereby maintaining formerly established genetic breaks.

### Genetic imprint and simulated dispersal trajectories

Plant propagules are likely to arrive at more remote locations through single dispersal events (Crisp et al., 2011). LDD events, by nature, remain difficult to observe, trace, or predict, both technically and conceptually (Jordano, 2017). Long-range dispersal events can be viewed as mutations (for the purpose of reasoning: it is about introducing an unrelated allele in a receiving host population). We did not observe a disjunction in allele distribution so we may conclude that LDD beyond subbasins is either a rare event or left undetectable. When considering the modeled trajectories, most particles leaving the Balearic Islands and Sardinia/Sicily in the Tyrrhenian Sea moved away from coastlines but potentially may reach other islands or coastlines after 2–4 weeks rafting. Results of genetic and physical connectivity assessments are in good agreement in this study on *Ruppia*, both confirming a strong isolation of most lagoons (cf., also for *Zostera noltii*; Jahnke et al., 2016), but also indicating that LDD is possible. Some gene pools are shared in the Balearic-Tyrrhenian and in Tyrrhenian-Ionian Sea. Both genetic structure and simulated dispersal trajectories indicated a unidirectional route from the Balearic to Tyrrhenian Sea and from the Western to the Eastern Mediterranean basin, thereby lowering their strength of an IBD. On the contrary, a stronger IBD in Baltic-Atlantic and Adriatic-Ionian Sea thus can be indicative of less mixing over long distances.

A study on *Posidonea oceanica* in basins of the Mediterranean Sea showed that breaks in connectivity could not be explained by contemporary currents, but that these patterns are confounded by historically deep vicariance (Serra et al., 2010). Contemporary ocean currents and historical isolation due to Pleistocene sea level fluctuations have been predicted to influence the genetic structure in marine and coastal populations (Hernawan et al., 2017). Throughout the Pleistocene (about 2.6 Ma-12 ka), sea level fluctuated following repeated glacial-interglacial cycles. About twenty thousand years ago, at the LGM, sea level in the Mediterranean was between 120 and 130 m below present-day sea level, changing the configuration of coastlines and water bodies, ocean currents, and the availability of habitats. Sea level rise went fairly rapid with increases of 3.7–2.5 m per century, 14,000 and 12,000–11,000 years ago, respectively (Collina-Girard, 2003). When sea level rose at the onset of the Holocene (about 11 ka), individuals from the Southern Adriatic/Ionian Sea could have colonized the exposed Northern Adriatic coastline (Triest and Sierens, 2014) and from the SW Atlantic moved to the North Sea and beyond the shallow continental shelve toward the Baltic (= post-LGM colonization). Very different from the Atlantic pattern of rapidly changing land, ice, saltwater and freshwater, the populations along Mediterranean coastlines most likely were moved “upward” and “landward,” thereby keeping the positions at major estuaries within subbasins and potentially maintaining an ancient genetic imprint. In more recent times, ocean currents along the Atlantic and toward the Baltic Sea (Johannesson and André, 2006) have contributed to patterns of connectivity along newly exposed habitats during last millennia and hundreds of years, particularly for coastal species with passive dispersal mechanisms and water as their main dispersal vector.

While the isolating/facilitating effect of contemporary processes such as water currents in seagrass connectivity is evident from experimental studies (Ruiz-Montoya et al., 2015), an analysis of *Thalassia hemprichii* showed that the signature of contemporary currents is less obviously reflected in the genetic patterns than the historical processes. The genetic imprint of more ancient processes was still strong in the population structure of *Thalassia hemprichii* of the Indo-Australian archipelago and Sunda shelf (Hernawan et al., 2017). *Thalassia* is adapted to floating seed dispersal whereas the most marine species of *Ruppia* has no adapted seeds but occasional detached shoot fragments. The actual persistence of previously differentiated genetic clusters in the Mediteranean subbasins, implies that contemporary gene flow is sufficiently restricted to maintain a more ancient differentiation. The pattern of genetic structure and connectivity of *Ruppia* in the Mediterranean thus can be attributed partly to the Pleistocene level fluctuations, only modified to a lesser extent by contemporary ocean currents. We put forward the hypothesis that the pre- and post LGM genetic imprint of *R. cirrhosa* along the European coasts was maintained by their occurrence in primary habitats, namely open lagoons, often with *Zostera* and seaweeds on sandy substrates. Secondary habitats are abandoned salinas, diked wetland areas of former lagoon systems (closed systems on muddy substrates without *Zostera* or seaweeds but with *Potamogeton pectinatus* L. or *Zannichellia pedunculata* Rchb.). The latter habitats with fluctuating salinities, often ephemeral, are also populated by *R. maritima* s.s., a Mediterranean taxon of hybrid origin, hybrids between *R. cirrhosa* and *R. maritima* and *R. drepanensis* (Triest and Sierens, 2014).

Dispersal of fruits and seeds by waterfowl, either through ingestion or adhesion to feathers were suggested to facilitate long distance movement of genes (Figuerola and Green, 2002; Santamaria, 2002; Higgins et al., 2003). Birds are mentioned to transport vegetative fragments and seeds of *Ruppia* and *Zostera* over hundreds of kilometers and potentially across continents (McMahon et al., 2014). However, reports of so-called LDD across continents (e.g., between Japan or China to Australia as reported by Ito et al., 2010 and Yu et al., 2014) are not at all supported by genetic data, but merely are inferences of a wide geographic distribution during the Pleistocene (Triest and Sierens, 2014; Triest et al., 2018). One should remain cautious with generalizations in *Ruppia*. The genus is more diverse in evolutionary significant units than previously thought (Triest et al., 2018). Records of species occurrences and publications are often based on misidentification, thereby calling many populations on different continents either *R. maritima* or *R. cirrhosa* (as argued in Triest and Sierens, 2014, 2015; Yu and den Hartog, 2014; den Hartog et al., 2016). We did not collect (nor find) the autotetraploid *R. cirrhosa* in truly inland brackish water populations where one typically finds, in ephemeral habitats, species such as *R. maritima*, hybrids with *R. cirrhosa, R. drepanensis* or another yet undescribed Mediterranean taxon, which have previously been reported to have larger amounts of fruits on their shoots than *R. cirrhosa* (Triest and Sierens, 2009, 2014, 2015). This potentially causes difficulties in identification of ESUs and hence estimating levels of connectivity. Water birds were shown to be main dispersal vectors of the continental *R. maritima* (Figuerola et al., 2002), but most probably refers to another taxon than *R. maritima* s.s. (Triest and Sierens, 2013, 2015; Triest et al., 2018) and other *Ruppia* species from ephemeral habitats that bear many seeds per plant (Rodriguez-Perez and Green, 2006). Water birds surely are responsible for genetic connectivity over relevant short distances of intense bird visits. Such events, if effective, are expected to confound other processes. When considering comparable bird movements and seed dispersal events along coastlines, then one should find comparable patterns of genetic connectivity. We however found strong barriers to genetic connectivity (also confirmed by DAPC) over short distances that strikingly coincide with modeled dispersal trajectories along coastal waters (i.e., Atlantic populations in SW France; SW Iberian populations south of the Balearic Sea, E and S Sardinia). Alternatively, our study showed connectivity and genetic mixtures within the Balearic Sea and connectivity between S Sardinia and Sicily and among Ionian lagoons. This broad-scale congruence between sea currents and barriers of connectivity merit further studies. In particular, a detailed comparison of the apparently high level of connectivity along Ionian lagoons with the low connectivity along the SE Iberian coastline could be interesting to estimate the relative importance of large-scale ancient imprint remainders vs. local bird-mediated dispersal.

A strong population genetic structure and isolation was observed in a regional study of the Southern Iberian Peninsula (Martínez-Garrido et al., 2017) including sites on few stretches of both the Atlantic and Mediterranean coastline. At that regional scale, bird-mediated dispersal (straight flight distances) appeared more significant as could be derived from Mantel tests using shortest coastal paths (not currents) and population distances (Martínez-Garrido et al., 2017). However, one should remind that a Mantel test can be strongly influenced by repeated sampling sites within a same lagoon, thereby enhancing the *r*^2^ because of low differentiation over shortest spatial (within-lagoon) distances, e.g., few km between three vegetation beds at northern edges of Mar Menor and within two beds of Cadiz Bay in the abovementioned regional study.

Both abiotic and biotic vectors are expected to confound each other and unraveling their relative importance from genetic data depends on the time scale and spatial scale considered. The observed genetic structure of *R. cirrhosa* in Europe refers to a remnant of Pleistocene distribution or LGM imprint and contemporary post-LGM sea currents, whereas regional biotic vectors did not left clear traces of strict sense LDD. During the last 5 years, there was a renewed interest on *Ruppia* with many new findings on local, regional and continental scale that will form the basis for future studies beyond explorative phases. An experimental approach using informative molecular markers and a well-structured hierarchical sampling design at small and large spatial scales will be required to further unravel the relative importance of abiotic and biotic processes and their complex interactions on dispersal along different stretches of coastlines.

In conclusion, this study combined genetic analysis, threshold estimations of connectivity (CRT) and oceanographic modeling to understand mechanisms of dispersal and to give insight into species life-history traits of *R. cirrhosa*. We showed that contemporary dispersal among most populations is limited to within lagoon areas (5–20 km) or might remain below detection sensitivity. However physical and genetic methods detected limited connectivity from congruent threshold levels among populations at a distance of up to tens to a hundred of kilometers, which can be regarded as an ecologically significant scale for putative passively dispersing rafting of *R. cirrhosa* fruiting shoots. We showed for *R. cirrhosa* that oceanographic features may play an important role in shaping the overall historical connectivity of primary habitats. Biotic vehicles of transport (waterfowl) might interfere at a certain spatial scale and in secondary habitats, though not explaining the obtained genetic breaks and clustering of gene pools, when overlaid to modeled dispersal trajectories of ocean currents. The understanding of abiotic and biotic interactions of submerged plants, their pollination mode, dispersal vectors and habitat suitability benefit from a combination of genetic and hydrodynamic modeling to improve our understanding of connectivity and consequences of movement in coastal lagoons and wetlands.

## Author contributions

LT performed the fieldwork, conceived and designed the experiments, contributed reagents, materials, analysis tools, interpreted, and analyzed the genetic data, wrote the paper, prepared tables. TS performed and interpreted the microsatellite experiments. TVdS and DM carried the modeling component of this research at the Jet Propulsion Laboratory (JPL), California Institute of Technology (CalTech), TVdS under a JPL Visiting Student Researchers Program (JVSRP), and DM under a contract with the National Aeronautics and Space Administration (NASA). TVdS prepared figures. TVdS and DM reviewed drafts of the paper.

### Conflict of interest statement

The authors declare that the research was conducted in the absence of any commercial or financial relationships that could be construed as a potential conflict of interest.
