# Supplementary material for: Inferring Connectivity Range in Submerged Aquatic Populations (Ruppia L.) Along European Coastal Lagoons From Genetic Imprint and Simulated Dispersal Trajectories
Source: Front Plant Sci. 2018 Jun 13;9:806. doi: 10.3389/fpls.2018.00806 (PMC6008504; doi:10.3389/fpls.2018.00806)
Supplement: Supplementary file 1 [file Table_1.DOCX]

**Supplementary Table S1** | **Microsatellite characteristics with range of allele sizes as encountered in 46 populations and detailed features of four populations (codes as in Table 1).**

| Locus | Primer sequences 5'-3' (and dye) | Genbank no. | Repeat motif | Allele sizes | Population | *A* | *A*_R_ | *H*_E_ | *H*_O_ |
| --- | --- | --- | --- | --- | --- | --- | --- | --- | --- |
| *RC3* | PET-ACCCATTTTTCTGGCCTTCT | KX758407 | (AG)14AA(AG)9 | 159-233 | 3_GER | 10 | 5.9 | 0.763 | 0.787 |
|  | R: GATACCAACCGCTTTTTCCA |  |  |  | 13_FR | 16 | 9.6 | 0.872 | 0.889 |
|  |  |  |  |  | 29_IT | 14 | 8.8 | 0.862 | 0.889 |
|  |  |  |  |  | 37_GR | 8 | 6.6 | 0.816 | 0.950 |
| *RCS27* | 6FAM-CATGGCTTCCTAATTGCTATAGAT | KX758408 | (TC)21 | 71-125 | 3_GER | 5 | 3.4 | 0.584 | 0.640 |
|  | R: GCTAGCAGGCAGTTGATGTTC |  |  |  | 13_FR | 10 | 7.4 | 0.813 | 0.926 |
|  |  |  |  |  | 29_IT | 8 | 4.8 | 0.641 | 0.630 |
|  |  |  |  |  | 37_GR | 4 | 2.4 | 0.273 | 0.250 |
| *RCS5* | NED-GAGGAGCGGGTGTTACAGAA | KX758409 | (AG)7 | 99-117 | 3_GER | 4 | 3.9 | 0.712 | 0.529 |
|  | R: TCTCTTTCTCTTCGCATTCTTG |  |  |  | 13_FR | 6 | 4.5 | 0.703 | 0.852 |
|  |  |  |  |  | 29_IT | 6 | 4.8 | 0.708 | 0.556 |
|  |  |  |  |  | 37_GR | 2 | 2.0 | 0.258 | 0.100 |
| *RCS8* | PET-TCTCTTCTTTCTCATTCTCTATTCG | KX758410 | (TCTT)8 | 97-129 | 3_GER | 3 | 2.5 | 0.525 | 0.480 |
|  | R: AGAAGCAAATCAAGCAGGAC |  |  |  | 13_FR | 4 | 2.9 | 0.573 | 0.556 |
|  |  |  |  |  | 29_IT | 7 | 4.7 | 0.713 | 0.630 |
|  |  |  |  |  | 37_GR | 4 | 3.7 | 0.706 | 0.800 |
| *RCS9* | VIC-TTCAGATTTCTCTTACTTGCTTTCG | KX758411 | (TTC)9 | 90-126 | 3_GER | 2 | 1.9 | 0.272 | 0.240 |
|  | R: CCAAGCTGACAACAATACGAGTT |  |  |  | 13_FR | 4 | 3.8 | 0.693 | 0.815 |
|  |  |  |  |  | 29_IT | 3 | 2.2 | 0.524 | 0.296 |
|  |  |  |  |  | 37_GR | 2 | 2.0 | 0.381 | 0.400 |
| *RM12* | 6FAM-TGTCAACCCACCACTCTTGA | KX758412 | (GAA)4+(TC)25 | 207-227 | 3_GER | 8 | 5.2 | 0.629 | 0.667 |
|  | R: CTCCTCGACAACCTCTCCTG |  |  |  | 13_FR | 5 | 4.0 | 0.620 | 0.926 |
|  |  |  |  |  | 29_IT | 5 | 3.9 | 0.556 | 0.560 |
|  |  |  |  |  | 37_GR | 2 | 2.0 | 0.454 | 0.400 |
| *RM26* | 6FAM-CTCTGTTAAGGCCCTTGCTG | KX758414 | (GA)11 | 176-218 | 3_GER | 10 | 7.4 | 0.846 | 0.760 |
|  | R: GCATTTGATTCTTTCCCTTCTC |  |  |  | 13_FR | 8 | 6.5 | 0.848 | 0.667 |
|  |  |  |  |  | 29_IT | 6 | 5.7 | 0.825 | 0.188 |
|  |  |  |  |  | 37_GR | 4 | 4.0 | 0.735 | 0.333 |
| *RM3* | VIC-CGGAACGTCAACGTTAGGAG | KX758416 | (AG)4GG(AG)3 | 223-245 | 3_GER | 2 | 2.0 | 0.451 | 0.500 |
|  | R: GTTAACCCCACCAGCCTCTT |  | +(AT)5GC(AG)9 |  | 13_FR | 3 | 2.5 | 0.468 | 0.538 |
|  |  |  |  |  | 29_IT | 4 | 3.7 | 0.647 | 0.450 |
|  |  |  |  |  | 37_GR | 5 | 4.5 | 0.694 | 0.667 |
| *RMB15* | VIC-GTGGACGTAGGCCAAGTTGT | KP090406 | (TG)10 | 161-185 | 3_GER | 3 | 2.7 | 0.394 | 0.458 |
|  | R: TTTCTCGCTCCTTTGGAAAA |  |  |  | 13_FR | 7 | 4.9 | 0.717 | 0.704 |
|  |  |  |  |  | 29_IT | 8 | 6.6 | 0.825 | 0.852 |
|  |  |  |  |  | 37_GR | 6 | 4.6 | 0.586 | 0.700 |
| *RUMR4* | ACTAAGTACTCCTCGAATCA | GQ246488 | (CT)2CC(CT)8 | 122-138 | 3_GER | 2 | 2.0 | 0.506 | 0.560 |
|  | R: ATCAATGGTGTTTGTATGGT |  |  |  | 13_FR | 2 | 2.0 | 0.406 | 0.407 |
|  |  |  |  |  | 29_IT | 4 | 2.5 | 0.458 | 0.407 |
|  |  |  |  |  | 37_GR | 2 | 2.0 | 0.505 | 0.700 |

*Populations 3_GER (N = 25), 13_FR (N = 27), 29_IT (N = 27) and 37_GR (N = 20) with number of alleles (A), Allelic richness (A_R_) at k=20 gene copies, gene diversity corrected for sample size (H_E_), and observed heterozygosity (H_O_*)*.*
