# Supplementary material for: Inferring Connectivity Range in Submerged Aquatic Populations (Ruppia L.) Along European Coastal Lagoons From Genetic Imprint and Simulated Dispersal Trajectories
Source: Front Plant Sci. 2018 Jun 13;9:806. doi: 10.3389/fpls.2018.00806 (PMC6008504; doi:10.3389/fpls.2018.00806)
Supplement: Supplementary file 2 [file Table_2.DOCX]

**Supplementary Table S2 | Detailed results of BAPS group level mixture analysis using K2-K46.**

Number of clustered groups: 46

Number of clusters in optimal partition: 35

Log(marginal likelihood) of optimal partition: -30424.8889

Best Partition:

Cluster 1: {23_SP, 24_SP} = hydrologically connected neighbouring sites in Menorca

Cluster 2: {41_GR, 42_GR} = hydrologically connected neighbouring sites in Greece

Cluster 3: {2_GER, 3_GER, 4_GER} = hydrologically connected neighbouring sites in Hiddensee, Germany

Cluster 4: {37_GR}

Cluster 5: {35_SLO}

Cluster 6: {36_SLO}

Cluster 7: {12_FR, 13_FR} = hydrologically connected neighbouring sites in Camargue, France

Cluster 8: {14_FR}

Cluster 9: {39_GR}

Cluster 10: {25_IT}

Cluster 11: {26_IT}

Cluster 12: {5_GER}

Cluster 13: {32_IT, 33_IT} = hydrologically connected neighbouring sites in Trapani, Sicily, Italy

Cluster 14: {34_IT}

Cluster 15: {10_FR}

Cluster 16: {11_FR}

Cluster 17: {15_SP}

Cluster 18: {16_SP, 17_SP} = neighbouring sites in Estartit, Spain (new pond next to coastal river population)

Cluster 19: {20_SP}

Cluster 20: {31_IT}

Cluster 21: {38_GR, 40_GR} = hydrologically connected neighbouring sites in Arta lagoon, Greece

Cluster 22: {28_IT}

Cluster 23: {18_SP}

Cluster 24: {29_IT}

Cluster 25: {45_GR, 46_GR} = hydrologically connected neighbouring sites in Greece

Cluster 26: {47_GR}

Cluster 27: {43_GR, 44_GR} = hydrologically connected neighbouring sites in Greece

Cluster 28: {6_NL}

Cluster 29: {19_SP}

Cluster 30: {22_SP}

Cluster 31: {30_IT}

Cluster 32: {21_SP}

Cluster 33: {27_IT}

Cluster 34: {7_FR, 8_FR} = neighbouring sites in Nord-Pas-de Calais, France (coastal duck ponds)

Cluster 35: {9_FR}

List of sizes of 10 best visited partitions and corresponding log(ml) values

35 -30424.8889

34 -30424.9235

36 -30426.3779

35 -30428.7166

34 -30428.7512

35 -30430.0091

34 -30440.1957

36 -30440.9827

35 -30441.0174

37 -30442.4717

Probabilities for number of clusters

34 0.44056

35 0.45874

36 0.1007
